# Melatonin: Regulation of Prion Protein Phase Separation in Cancer Multidrug Resistance

**DOI:** 10.3390/molecules27030705

**Published:** 2022-01-21

**Authors:** Doris Loh, Russel J. Reiter

**Affiliations:** 1 Independent Researcher, Marble Falls, TX 78654, USA; 2 Department of Cellular and Structural Biology, UT Health San Antonio, San Antonio, TX 78229, USA

**Keywords:** melatonin, prions, cancer multidrug resistance, tumor microenvironment, liquid–liquid phase separation, hypoxia, pH, heme iron, band 3, copper

## Abstract

The unique ability to adapt and thrive in inhospitable, stressful tumor microenvironments (TME) also renders cancer cells resistant to traditional chemotherapeutic treatments and/or novel pharmaceuticals. Cancer cells exhibit extensive metabolic alterations involving hypoxia, accelerated glycolysis, oxidative stress, and increased extracellular ATP that may activate ancient, conserved prion adaptive response strategies that exacerbate multidrug resistance (MDR) by exploiting cellular stress to increase cancer metastatic potential and stemness, balance proliferation and differentiation, and amplify resistance to apoptosis. The regulation of prions in MDR is further complicated by important, putative physiological functions of ligand-binding and signal transduction. Melatonin is capable of both enhancing physiological functions and inhibiting oncogenic properties of prion proteins. Through regulation of phase separation of the prion N-terminal domain which targets and interacts with lipid rafts, melatonin may prevent conformational changes that can result in aggregation and/or conversion to pathological, infectious isoforms. As a cancer therapy adjuvant, melatonin could modulate TME oxidative stress levels and hypoxia, reverse pH gradient changes, reduce lipid peroxidation, and protect lipid raft compositions to suppress prion-mediated, non-Mendelian, heritable, but often reversible epigenetic adaptations that facilitate cancer heterogeneity, stemness, metastasis, and drug resistance. This review examines some of the mechanisms that may balance physiological and pathological effects of prions and prion-like proteins achieved through the synergistic use of melatonin to ameliorate MDR, which remains a challenge in cancer treatment.

## 1. Introduction

The symptom of prion protein infection was first described in 1732 when Merino sheep scraped pathologically against fences [1], but the term prion (PRoteinaceous Infective ONly particle) was not coined until 1982 by Prusiner who defined prions in 1998 as heritable, infectious, proteinaceous particles that are converted from the normal, cellular form (PrP^C^) into the pathogenic form (PrP^Sc^) that associates with amyloid plaques [2,3]. The full-length prion protein (PrP) [4] exists as a native, soluble cellular PrP^C^ isoform with important physiological functions [5] including cellular differentiation [6,7,8], proliferation [9], and adhesion [10]; myelin maintenance [11]; circadian rhythm regulation [12,13]; signal transduction [14]; glucose homeostasis [15,16]; immune regulation [17,18]; as well as copper homeostasis, utilization [19,20]; iron uptake, transport, and metabolism [21,22,23]; and even facilitating the persistence and storage of memory [24,25]. In humans, quantitative transcriptomics analysis (RNA-Seq) of 27 different tissues obtained from 95 human individuals [26] found the prion gene *PRNP* to be ubiquitously expressed in all 27 human tissues examined in addition to mitochondria, with the highest expressions found in the brain, followed by the ovary, prostate, heart, gallbladder, endometrium, adrenal, urinary bladder, thyroid, testis, skin, esophagus, and lung [27]. Cellular PrP^C^ has since been identified in brain mitochondria of wild-type and transgenic mice in the absence of disease [28]. After Masison and Wickner discovered the prion protein in *Saccharomyces cerevisiae* [29], increased understanding of prion physiological and pathological functions began to converge on the “prion hypothesis”, where non-Mendelian, protein-based, epigenetic inheritance in prions is proposed to be the essential driving force behind prion propagation [30,31,32,33,34,35,36,37,38,39].

Prion-based inheritance of conformationally-encoded phenotype information may allow genetically identical cells to express diverse, adaptive phenotypes with distinct evolutionary advantages [39,40,41,42]. The study of prions in yeast reveals a unique “bet-hedging” feature [43,44] where cells form reversible prion colonies that can readily adapt to changing stress conditions in the environment. Cells with phenotypes created by prions may survive with a fitness advantage that is lost in cells without prions. Prion proteins allow yeast cells to adapt instantaneously to changing environments where frequency of phenotype gain/loss is dictated by the level of stress in the environment [45]. The fact that prions are often overexpressed in invasive, drug-resistant cancers highlights the important connection between the “prion hypothesis” and cancer MDR [46].

Tumor cells adapt to stressful environmental pressure including anti-cancer therapies by remodeling signaling pathways involving transcription, translation, and posttranslational modifications [47]. Tumor heterogeneity and plasticity are formidable challenges to overcome in drug resistance [48]. Reversibility of phenotypes in both cancer cells and prions allows the speedy addition or removal of genetic traits as adaptations to environmental stress [49]. It is perhaps not a coincidence that the spontaneous phenotype shifts in a highly metastatic murine fibrosarcoma cell line (KHT), observed to be approximately 10^−5^ per cell per generation [50], matches the 10^−5^ per cell per generation frequency of phenotype alterations from de novo prion formation reported in haploid *S. cerevisiae* strains [51]. Even though phenotype alterations may be reversible in both metastatic melanoma cells [52] and yeast prions [44,53], the reversible “curability” of [URE3]—the prion form of *Ure2* protein in haploid yeast first observed by Wickner in 1994—was actually a reflection of the temporary inactivation by guanidinium (a curing agent) of the conversion of *Ure2* into the altered [URE3] prion form, which then promptly repopulated itself under selective conditions [30]. Mutations or overexpression of *Ure2* can increase the conversion into prion [URE3] by 1000-fold [30,51].

Prions are often overexpressed in many forms of cancer [54,55,56], and the prion protein gene (*PRNP*) was detected by means of in silico analysis to be mutated in some cancer patients [57]. The ability of prions to enhance cancer proliferation, invasion, metastasis, increase stemness, and promote resistance to cytotoxic therapeutics has been extensively reviewed [55,58,59,60,61,62,63,64,65,66,67,68,69,70,71]. Since prion expression and conversion from the normal, soluble state to the pathogenic, aggregate form can be induced by stress [72], it is not surprising that prions are associated with MDR in many types of cancer [73,74,75] including gastric cancer [76], breast cancer [77], glioblastoma multiforme [78], and colorectal cancer [79], whereas silencing prion protein expression re-sensitizes breast cancer cells to adriamycin [80] and colorectal cancer cells to fucoidan [81]. Results from two randomized trials that evaluated the expression of PrP^C^ protein in normal breast and breast cancer tissues from 756 ER-negative breast cancer patients revealed a significant correlation with resistance to adjuvant chemotherapy in ER-negative disease [82]. The overexpression of PrP^C^ in cancer may be an innate, adaptive response conferring survival advantage reflecting evolutionary selection pressure [46,83].

Many anticancer drugs including cisplatin [84,85], doxorubicin [86], and temozolomide [87] exert their oncostatic efficacy by elevating production of reactive oxygen species (ROS) to enhance oxidative damage. The fact that PrP^C^ enhances clinical resistance to cisplatin in colorectal cancer cell [88] and increases invasiveness and resistance to doxorubicin-induced apoptosis in LS 174T colon cancer cells [89] supports the theory that PrP^C^ serves important physiological functions [5] including antioxidant protection [90]. PrP^C^ was observed to defend against ROS-induced DNA damage in human neuroblastoma SH-SY5Y cells [91] while 1C11 cells from PrP^null^ C57BL/6J mice displayed a 50% increase in ROS combined with a reduction in major antioxidant systems, including reduced glutathione (GSH) [92]. Some of the antioxidant effects associated with PrP^C^ depend on its ability to bind metal ions such as copper. The octarepeat peptide region in the unstructured N-terminal domain of PrP^C^ contains histidine residues that possess high binding affinity to copper and can form complexes with doxorubicin to significantly lower drug efficacy [93,94]. PrP^C^ interaction with temozolomide in glioma resulting in drug resistance may also be related to copper-binding effects [95,96].

The study of melatonin as an anti-cancer adjuvant [97,98,99] and oncostatic agent capable of inhibiting cancer metastasis while enhancing drug efficacy has been extensively documented and reviewed [100,101,102,103,104,105,106,107,108,109,110,111,112]. However, the interactions between melatonin and prions in cancer are not straightforward. For example, melatonin upregulates PrP^C^ expression to protect mesenchymal stem cells (MSCs) against ischaemic injury [113] but inhibits PrP^C^ expression to cause apoptosis in colorectal cancer cells [114]. When used in combination with anti-cancer drugs such as oxaliplatin and 5-fluorouracil (5-FU), melatonin becomes even more effective in inducing apoptosis and senescence in 5-FU-resistant colon stem cells and oxaliplatin-resistant colorectal cancer cells by suppressing PrP^C^ expression [115,116]. Yet melatonin was also reported to protect MSCs harvested from chronic kidney disease (CKD) mouse models against H_2_O_2_-induced senescence by upregulating PrP^C^ expression [113].

The seemingly contradictory, pleiotropic interactions between melatonin and prions actually reflect a delicate balance pivoted upon their intrinsic, natural response to stress. PrP^C^ has recently been demonstrated to protect animals from acute, inflammatory lipopolysaccharide (LPS, *Escherichia coli* O26:B6) challenge by modulating the expression of immune response genes [117]. Similarly, in MSCs treated with LPS-stimulated macrophages, the addition of 1 μM (but not 0.1, 10, or 100 μM) melatonin upregulated PrP^C^ expression and produced a maximal effect in conferring resistance against oxidative stress by enhancing MSC proliferation [118]. Conversely, using 2 mM melatonin (2000-fold increase) in LPS-stimulated prostate cancer cells inhibited migration and invasion [119]; the addition of 1 mM melatonin also inhibited cellular prion protein expression to promote apoptosis via superoxide-mediated oxidative stress in colorectal cancer cells [114]. It is plausible that at appropriately high concentrations, melatonin modulates the inhospitable, highly stressful tumor microenvironment (TME) to attenuate elevated PrP^C^ stress responses that may activate the conversion into pathological, self-templating aggregates, whereas lower levels support the natural, physiological protective reactions of prions under duress. The conversion of PrP^C^ into self-templating aggregates is now believed to be associated with liquid–liquid phase separation (LLPS), which is an energy-efficient thermodynamic process that results in the rapid formation and dissolution of biomolecular condensates used by living organisms as adaptation to changing environments [120,121,122,123,124]. Living organisms may have always relied upon melatonin to effectively modulate prion propagation using unique features including the regulation of liquid–liquid phase separation [125].

## 2. Liquid–Liquid Phase Separation May Regulate Prion Conversion and Propagation

Melatonin (N-acetyl-5-methoxytryptamine) is extensively studied for its potent antioxidant cascading reactions which continue to generate effective free radical scavenging metabolites while interacting with different ROS [126,127,128,129,130,131,132,133,134,135]. Since its discovery in the bovine pineal gland in 1958 [136], melatonin is now understood to be mainly produced in mitochondria in all present-day vertebrates [137,138,139]. The early, successful distribution of melatonin via horizontal gene transfers may accentuate the preponderant reliance on this ancient molecule for protection against endogenous and exogenous stress in all eukaryotes and bacteria tested [140,141,142,143,144]. Similar to the induction of prions in yeast as a response to stress, unfavorable exogenous or endogenous conditions such as oxidative stress, nutrient deprivation, and fluctuations in temperature and pH also induce increased production of melatonin in plants [145,146,147,148] and animals [149,150,151,152,153]. It has been proposed that a high reserve/maximum capacity of melatonin synthesis in humans provides a higher level of survival fitness as effective adaptation to unpredicted internal and external environmental stressors while enhancing recovery rates from injury and external pathogenic attacks [154,155]. Since *S. cerevisiae* can produce varying levels of melatonin under different nutritional and environmental conditions [156,157,158] and absorb exogenous melatonin in an oxidative stress-dependent manner [159,160], it is possible that living organisms may have evolved the ability to adjust appropriate levels of melatonin during stress exposure to support PrP^C^ physiological functions while restraining pathological conformational changes as part of stress adaptation including exposure to lethal doses of ultraviolet (UV) irradiation.

### 2.1. Melatonin May Modulate Stress-Induced Prion Conversion

The pathological prion PrP^Sc^ isoform is extremely resistant to inactivation by UV radiation at 254 nm with doses up to 120,000 J/m^2^ [161,162]. Cellular PrP^C^ expression is often increased in neuroblastoma, breast, and colorectal cancer cell lines after ionizing radiation treatment, and suppressing PrP^C^ can reduce radioresistance in tumor cells [163]. *S. cerevisiae* pretreated with melatonin were enriched in a dose-dependent manner and were protected from H_2_O_2_-induced oxidative stress as well as exposure to 254 nm UV irradiation with increased cell viability via dynamic modulation of antioxidant genes [160]. Even though melatonin is radio-protective [164], it can also increase radiosensitivity when used as adjuvant with radiotherapy to substantially improved tumor remission, 1-year survival, and alleviation of radiochemotherapy-related cytotoxic effects [61,165,166] such as the increased expression of heat shock protein 70 (Hsp70) [167,168]. Exposure to UV irradiation has been reported to elevate Hsp70 in yeast, human skin cells, murine fibroblasts and keratinocytes, as well as transgenic mouse models. Increased expression of Hsp70 is generally considered as protective [169,170,171,172], but the induction of Hsp70 also leads to increased expression of PrP^C^ as part of the adaptive antioxidant responses.

Melatonin is a potent antioxidant that is produced in human skin, and UVB irradiation of human keratinocytes resulted in intensely elevated local melatonin metabolism that was dependent and directly proportional to UVR dose applied [173]. The use of 1 mM melatonin prevented DNA damage and suppression of antioxidant enzymes and proteins in UVR-treated ex vivo human skin [174]. More importantly, melatonin suppressed the upregulation of Hsp70 in human full-thickness skin and human epidermal keratinocytes exposed to UV radiation but complemented the suppression of Hsp70 by reversing all effects induced by Hsp70 inhibition such as enhanced gene expression of proinflammatory cytokines and proapoptotic proteins [175]. The suppression of Hsp70 by melatonin provides a glimpse into the complex interplay between melatonin and prions where living organisms may use melatonin as a “broad-based metabolic buffer” to tune prion propagation in response to stress.

Experimental studies on *S. cerevisiae* showed that excess Ssa1 of the Hsp70 family was responsible for the de novo formation of [PSI^+^] which is the pathological prion isoform of yeast release factor Sup35 [169]. Even though contradictory results were reported in a study where the use of 0.5 and 1.5 µM 17-(dimethylaminoethylamino)-17-demethoxygeldanamycin (17-DMAG) [176]—a semi-synthetic derivative of the antibiotic geldanamycin—strongly induced Hsp70 expression in a rabbit kidney epithelial (RK13) cell line but significantly decreased PrP^Sc^ accumulation, the authors also acknowledged the completely unanticipated in vivo results that showed an increase in PrP^Sc^ from interactions with normal brain homogenates (NBH) obtained from WT Hsp70^+/+^ control mice, whereas NBH substrates from Hsp-null (Hsp^−/−^) mice did not support the generation of any PrP^Sc^ [177]. The confounding observation may be explained by the fact that 17-DMAG is unable to induce Hsp70 at concentrations below 20 nM, but the half-maximal inhibitory concentration (IC_50_) at only 8 nM 17-DMAG could inhibit the formation of misfolded proteins and toxic aggregates in polyglutamine disorders such as Huntington’s disease [178]. Therefore, a 62.5 or 187.5-fold increase in the use of 17-DMAG may have suppressed PrP^Sc^ accumulation regardless of Hsp70 activation status. However, it is also possible that in vitro and in vivo results for 17-DMAG are totally different as 17-DMAG administered to sepsis-LPS animal models at 5 mg/kg increased expression of Hsp70, conferring antioxidant protection to increase survival rates [179] which may imply activation of PrP^C^ by Hsp70.

The exposure of human NT-2 cells to heat (42 °C) simultaneously increased mRNA levels for both Hsp70 and PrP protein [180]. Most importantly, irradiation of human prion proteins at 302 nm caused complete structural unfolding with rapid precipitation and specific structural conversion into soluble β-sheeted oligomers with characteristics similar to structurally destabilized species that often precede pathological isoform aggregation [181]. However, the precipitation formed during UV irradiation entirely blocked UV transmission, implying that the original intention of aggregate formation is instinctively protective in nature [181]. It is now widely accepted that all living organisms depend upon the formation of dynamic, membraneless compartments in response to environmental changes. The balance between reversible and irreversible aggregation of these condensates during the process of liquid–liquid phase separation (LLPS) may be the linchpin that defines the fine line that separates health from disease [182].

### 2.2. The Intrinsically Disordered Region in Prions Is Requisite for Liquid–Liquid Phase Separation, Cytoplasmic Inheritance, and Modulation of Pathological Conversion

Biomolecular condensates are intracellular membraneless organelles (MLOs) that compartmentalize and organize proteins, ribonucleic acids (RNAs), and other nucleic acids [183]. In response to continuously changing endogenous or exogenous conditions, all living organisms including eukaryotes [184,185], prokaryotes [186,187], and archaea [188,189] depend on LLPS as the primary driver to fuel the condensation or dissolution of MLOs in rapid, energy-efficient reactions such as stress response [190], signal transduction [191,192], redox balance [193], as well as genome expression, organization and repair [194]. The canonical yeast translation termination factor Sup35, responsible for catalyzing translation termination during growth, contains an evolutionarily conserved, intrinsically disordered prion N-terminal domain that can phase separate under stress to form protective, reversible biomolecular condensates [124,195,196] which can restore cell growth functions upon termination of stress. However, the intrinsically disordered N-terminal region of Sup35 can also phase separate to form irreversible heritable aggregates that are the prion isoforms [PSI^+^] responsible for generating heritable phenotypic variations as part of stress adaptation [124,197,198].

Proteins with intrinsically-disordered prion or prion-like domains, which are often highly enriched in nucleic acid binding proteins but may be prone to the formation of fibrillar assemblies, are widely conserved across evolution and are accepted to be the source for protein-based cytoplasmic inheritance essential in the formation of new, opportunistic, adaptive traits that ensure survival in hostile environments [39,196,199,200]. Within the *Saccharomyces* proteome, intrinsically disordered proteins capable of LLPS are often located in the nucleus and are involved in the regulation of transcription and cell signaling [201]. Thus, the formation of reversible condensates rather than irreversible aggregates in response to stress may have been a primary function of prions and prion-like domains that serve as stress sensors and adaptors. Sup35 in many yeast species actually do not have the ability to form [PSI^+^] prions [202,203]. By contrast, intrinsically disordered regions (IDRs) in prion-like domains, which easily phase separate and form dynamic condensates, are highly conserved across all three domains of life—eukaryotes, prokaryotes, and archaea—as well as viruses [204,205,206,207,208,209]. LLPS of IDRs in proteins enables the rapid formation of membraneless organelles without mechanical barriers but are distinctly segregated by chemical boundaries [210,211]. However, phase separation at its core is an entropically unfavorable thermodynamic process requiring a reduction or a negative change in global free energy enabled by energetically favorable multivalent protein–protein interactions that can offset energetic costs [183,212,213].

#### 2.2.1. The Role of ATP and RNA in Prion Phase Separation

Thermodynamic nonequilibrium processes, such as posttranslational modification (PTM) involving the hydrolysis of adenosine triphosphate, which can induce free energy changes of −7.3 kcal/mol, can facilitate the exchange of substrates and information between condensates in their native and droplet states during LLPS [214,215,216,217]. Adenosine triphosphate (ATP) at micromolar concentration provides free energy to fuel phase separation by generating supersaturation gradients inducing droplet segregation [212,218,219]. However, ATP can also function as a biological hydrotrope at physiological concentrations from ~2 to 8 mM, solubilizing abnormal, pathological aggregates often associated with neurodegenerative disorders [220,221,222,223,224]. ATP is a universal and specific biphasic modulator of LLPS in IDRs, altering physicochemical properties, conformation dynamics, assembly, and aggregation [225]. More recently, ATP has been proposed to be a kosmotropic anion behaving like a “biological aggregation inhibitor” that can increase protein stability and reduce thermal aggregation [223,226,227].

Experimental studies revealed that LLPS can promote the spontaneous conversion of human and mouse recombinant prion protein (rPrP) into the pathological PK-resistant PrP^res^ isoform without involving kinetic energy or seeding from PrP^Sc^. However, the conversion process was dependent upon interactions between the intrinsically disordered N-terminal domain and kosmotropic anions [121]. Incubating kosmotropic anions with recombinant PrP elevates protein stability, inducing misfolding into PrP^Sc^ amyloid-like aggregates [228]. Yet the efficiency of droplet formation via LLPS did not exactly match with the Hofmeister series [121,229]. It is possible that interactions between ATP, which has recently been proposed to be a kosmotropic anion [226], and RNA can vitally influence the outcome of LLPS of prions and prion-like domains. Recent experimental results reported the ratio between the negatively-charged ATP and RNA can affect aggregation and dissolution where ATP competitively binds to condensate-forming proteins in IDRs to inhibit RNA-driven phase separation of the proteins [230].

Ribonucleic acid (RNA)—a single-stranded molecule with alternating ribose and phosphate groups attached to adenine, uracil, cytosine or guanine bases—is an essential architectural component that can influence the composition and morphological outcome of condensate phases in LLPS [231], as well as regulate spatiotemporal distribution of MLOs by fine-tuning biophysical properties such as viscosity and internal molecular dynamics [232]. RNA modulates condensate formation during LLPS due to the high negative charge densities buried in the phosphate backbones. As a result, phase separation can be promoted by a low level of negatively charged RNA molecules interacting with positively charged proteins, whereas high levels of RNA may repel the same proteins to dissolve condensates [233,234]. In essence, the IDRs of prions and prion-like domains drive phase separation and the assembly of condensates while RNA can regulate the dynamics of those condensates [235], potentially stimulating conversion of PrP^C^ into PrP^Sc^ [236,237,238]. ATP is one of the four nucleotide monomers required for RNA synthesis [239,240]. The intricate relationship between ATP and RNA may extend as far back as the highly-debated “RNA world” [241,242,243] when ATP was an integral energy-providing component of a metabolic system composed of nucleic acid enzymes, which is believed to precede the evolution of ribosomal protein synthesis [244,245].

Prions and prion-like molecules have likely assumed central roles in early chemical evolutionary processes preceding the Last Universal Common Ancestor (LUCA), which eventually resulted in present-day living systems [246,247]. The ability of prions to efficiently replace their non-aggregate native state by assembling short peptides into β-sheet amyloid aggregates with high structural stability and resistance to hostile, extreme environments may have facilitated self-replication, catalytic activities, and analogical information transfer in protein-based, self-propagating, information-processing biomolecules in early life forms ~3.9 billion years ago [248,249,250]. The phase behavior of the prion-forming protein Sup35 PrD in yeast is critically modulated by stress. Exposing Sup35 PrD to 10 mM arsenite promoted droplet formation in 93% of treated cells, whereas only 30% of untreated cells formed droplets [251]. Although the formation of non-infectious, self-assembled macromolecular complexes represents a vital physiological function, these assemblies are highly susceptible to the formation of pathological aggregates that are now associated with cancer [252,253,254] and neurodegenerative disorders. Cancer is now recognized as a disease that may result from aberrant LLPS and aggregation of MLOs [252,255,256,257,258,259,260,261,262]. Wild-type human tumor suppressor protein p53 expressed in yeast undergoes LLPS induced by multivalent interactions between its intrinsically disordered N- and C-termini to form unstable droplets that dissolve quickly when stress is removed. However, when overexpressed, the p53 protein lost tumor-suppressing transcription ability, forming aggregates that behaved in a comparable manner to stable, heritable prions [263,264,265].

#### 2.2.2. RNA- and Copper-Binding Modulate the Conversion of PrP^C^ to PrP^Sc^

The regulation of prion functionality and conversion into toxic aggregates may be fundamentally propelled by LLPS [120,121,122,123], and the intrinsically disordered N-terminal region of the physiological PrP^C^ has been shown to be necessary and sufficient for LLPS of PrP [266,267]. Large nucleation barriers enable deep supersaturation that favors the formation of toxic aggregates in Sup PrD while kinetic barriers for the formation of dynamic intracellular condensates are easily breached by PTMs and changes in salt, pH, and temperature during LLPS [251,268,269]. Nevertheless, fluctuations in RNA concentrations can modulate prion aggregation in a bimodal, concentration-dependent manner where high protein to RNA ratios stimulate aggregation and low ratios suppress condensate formation. RNAs of different sources and lengths were reported to markedly alter rPrP aggregation in a concentration-dependent manner [270]. Even though prions are understood to reside in lipid rafts on plasma membranes [271,272], prions found in cytosol of neuronal and non-neuronal cells form ribonucleoprotein (RNP) complexes similar to membraneless RNA granules or chromatoid bodies containing mRNAs, and RNA proteins including the DEAD-box RNA helicase DDX6 and other non-coding RNA, small nuclear RNA, and microRNAs. The domain located between residues 30 and 49 in the intrinsically disordered N-terminal is necessary for the assembly of these PrP-RNP granules, which is believed to have important functions in RNA processing and posttranscriptional gene regulation, and are different from other cytosolic prion-containing aggresomes previously observed [273,274,275,276]. However, when overexpressed, cytosolic PrP in neurons can exhibit toxicity in certain cell populations [277].

PrP^C^ replication environment depleted of RNA gave rise to a completely new strain of PrP^Sc^ without changing PrP primary structure [278]. Mutations in residues can increase binding of RNA to specific sites in PrP^C^, facilitating the formation of a pincer motif that leads to the decay of the N-terminal α-helix, which is a requisite step in the hastened conversion of PrP^C^ to the toxic, infectious PrP^Sc^ isoform [279,280]. Experimental studies showed that mutant peptides may exhibit greater resistance to cancer drugs such as cisplatin as a result of weakened adduct binding affinity. Although increasing the cisplatin ratio to 2:1 facilitated adduct binding, it was still ineffective in preventing aggregation [281], whereas truncated variants of rPrP lacking octarepeat peptides in the N-terminal domain were less susceptible to aggregation [270]. In fact, neutralizing mutations can considerably reduce cytotoxicity from amyloid fibril formation in the prion-prone peptide PrP 106–126 belonging to the intrinsically disordered N-terminal domain [282]. Perhaps not coincidentally, MDR in gastric cancer is associated with four of the five copper-binding octarepeat peptides located within the N-terminal domain. Mutant gastric cancer cells constructed from gene splicing lacking octarepeat peptides (residues ~51–91) exhibited highly decreased anti-apoptotic capacity and lowered antioxidant responses to stress [283,284].

Prion protein antioxidant defense is specifically mediated by ROS cleavage and copper-binding in the octarepeat peptide region in the N-terminal domain [285,286,287]. Copper is an essential trace element used in all domains of life as a structural component for proteins and as cofactor in catalytic oxidation-reduction (redox) reactions that can result in the production of ROS [288]. Binding of copper (Cu(II), Cu^2+^) to PrP^C^ facilitates redox balance and copper homeostasis [289] both of which are often disturbed in the TME where cancer drug resistance is associated with higher serum copper levels in patients compared to healthy controls or patients who responded to chemotherapy [290,291]. Copper also changes the conformation of the N-terminal domain [292,293,294,295], which may impede LLPS [121,296] or even prevent the formation of straight β-strands backbone structures in the infectious PrP^Sc^ form when bound to the non-octarepeat peptides (residues 92–96) [297,298]. However, the Cu^2+^ inhibition of amyloid formation is dependent upon binding capacity that becomes less effective at a lower pH [93,299], which is characteristic of most TMEs. In addition, under physiological conditions, Cu^2+^ bound to full-length, uncleaved PrP can induce misfolding that increases seeding, which serves as templates for aggregation [300,301]. Melatonin is not only a potent antioxidant capable of chelating copper and modulating ROS-dependent prion cleavage, but is also proposed to be an important regulator of phase separation [125].

### 2.3. The Role of Melatonin in the Regulation of Liquid–Liquid Phase Separation and ROS-Induced Cleavage in Prions

Phase separation is an evolutionarily conserved response used by living organisms to assemble biomolecular condensates as efficient adaptation to rapidly changing endogenous or exogenous stressors [190,196]. The formation of condensates during LLPS is a process of nucleation and growth constrained by an energy barrier that can usually be breached by thermodynamic nonequilibrium PTMs [269,302]. Many well-known targets of melatonin including NLRP3 inflammasome [303,304,305] and tumor suppressor protein p53 [306,307,308] contain prion-like IDRs that facilitate LLPS [265,309,310,311] and are regulated by ATP-dependent PTMs such as phosphorylation, ubiquitination, and SUMOylation [312,313,314,315,316,317], while DEAD-box RNA helicases such as DDX3X, which are tuned by RNA and ATP [318], can critically determine the outcome of prionoid LLPS in NLRP3 [310]. Posttranslational modification of PrP^C^ initiates and/or propagates PrP^Sc^ aggregates [319,320], profoundly altering prion assembly pathways [321] to produce new strains with different protein conformations in vivo [322]. The addition of a single, fully-charge phosphate group at pH 7.5 to various locations in human peptide sequence corresponding to residues 59–71 from the intrinsically disordered N-terminal domain inhibited fibril formation, whereas phosphorylation of the same peptides at pH 1.1, when the phosphate is fully protonated, caused rapid fibril formation [323].

Melatonin may efficiently mediate important PTMs that regulate proteins which can form physiological condensates or pathological prion-like aggregates due to its ability to protect mitochondrial and cytoplasmic ATP levels and maintain requisite RNA concentration, which not only ensure proper formation and dissolution of condensates [125] but possibly also modulate reentrant phase transitions that are important biochemical timekeeping RNA-dependent transformations where increased RNA dissolves condensates to return to an identical or macroscopically similar state before the phase transition [324]. Since prion targeting of lipid rafts [272,325,326] can affect membrane signaling [327,328] and lipid composition [329], the role of melatonin in the prevention of lipid peroxidation, modification of lipid hydrocarbon chain to promote phase separation in ternary membrane models [330,331], stabilizing lipid liquid ordered (L_o_) to liquid disordered (L_d_) phase separation over a range of temperatures [332], and displacing cholesterol in competitive binding to lipid molecules [330] provides additional insight into the complex relationship between melatonin and prion physiological and potential pathological conversion mediated by phase separation and associated processes.

#### 2.3.1. The Role of Melatonin in PrP^C^ LLPS and Amyloid Beta Binding

**Figure 1:**
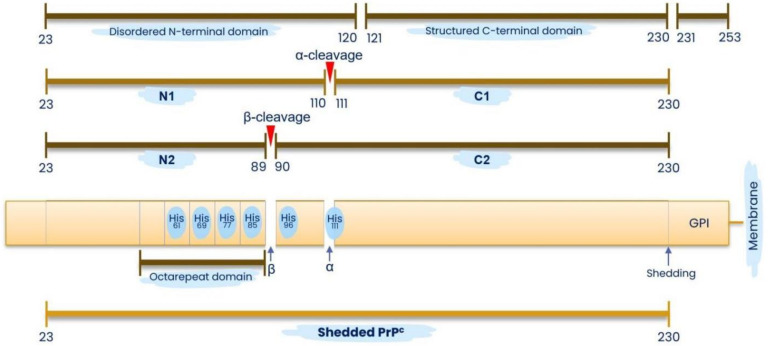
Schematic representation of the prion protein structure. Residues 1–23 comprise the N-terminal signal peptide that is cleaved upon maturation; residues 23–120 comprise the unstructured N-terminal domain; residues 121–230 comprise the structured C-terminal domain; and residues 231–253 comprise the GPI anchor signal tethered to lipid rafts on plasma membranes. α-cleavage of residues 110/111 yields N1 (residues 23–110) and C1 (residues 111–230) fragments while ROS-induced β-cleavage at residues 89/90 produces N2 (residues 23–89) and C2 (residues 90–230) fragments. Four histidine residues in the octarepeat domain and two histidine residues in the fifth nonoctarepeat binding site exhibit high-affinity to copper ions. Shedding by proteolysis of the GPI anchor at residues 230–231 releases a full-length, soluble PrP^C^ (23–230).

Cellular PrP contains 253 residues and is often bound to lipid rafts on membranes via glycosylphosphatidylinositol (GPI) anchors [272,333]. Residues 1 to 23 comprise the N-terminal signal peptide that is cleaved upon maturation; residues 23 to 120 comprise the positively charged, unstructured N-terminal domain; residues 121 to 230 comprise the structured C-terminal domain; and residues 231 to 253 comprise the GPI anchor signal [272,333,334,335] (Figure 1). PrP^C^ is rich in α-helical content and can be converted into insoluble, non-infections proteinase K (PK)-resistant (PrP^res^) isoforms [121,336,337] or infectious, stable, PK-resistant PrP^Sc^ isoforms, rich in aggregation-prone β-sheet structures that are associated with detrimental, cytotoxic effects [2,335,337,338,339,340]. Early workers postulated that the misfolding of PrP^C^ is the central mechanism governing the conversion to the toxic PrP^Sc^ isoform, and that the earliest event in prion misfolding involves metastable intermediates with aggregation-prone, β-sheet enriched structures [341,342,343,344].

Experimental studies in 2018 reported that PrP^C^ undergoes LLPS at physiological pH and salinity, and can exist in multiple phases with extensive secondary structure rearrangement. LLPS of PrP^C^ and N-terminal residues 23–110 (N1) could be triggered by excess amyloid-β oligomers (Aβo), resulting in the formation of reversible hydrogels with up to 300-fold Aβo enrichment. These Aβo/PrP hydrogels engaged signal-transducing metabotropic glutamate receptor mGluR5 and altered its cell surface mobility [345]. It was proposed that Aβo binding by soluble (anchor-free) prion protein and N-terminal fragments is a protective response [346] due to reports showing N1 strongly suppressed Aβo toxicity in vitro and attenuated Aβ-induced memory function in a mouse model in vivo, in addition to inhibiting the aggregation and assembly of Aβ(1–42) into amyloid fibrils, reducing neurotoxicity [347].

Aβo is a pathological ligand [348] often found to cluster at excitatory synapses with mGluR5 and PrP^C^, acting as a scaffold for mGluR5 to disrupt synaptic function and glutamate signaling [349,350,351]. The interactions between Aβo–PrP^C^ and mGluR5 at the neuronal surface also activate the cytoplasmic Fyn tyrosine kinase to undermine neuronal functions and plasticity via increased intracellular calcium [352,353,354,355]. Fyn is associated with cancer pathogenesis and drug resistance and is often found to be upregulated in prostate cancer [356] and tamoxifen-resistant breast cancer cell lines [357]. In gastric cancer, Fyn is frequently overexpressed and is positively correlated with metastasis [358]. Not surprisingly, PrP^C^ accelerates colon cancer metastasis via the Fyn-SP1-SATB1 axis [62]. The fact that melatonin can downregulate mGluR5 expression by diminishing Tet1 expression, which uncouples Tet1 from the mGluR5 promoter [359], modulating the PrP^C^/mGlur5/Fyn/Pyk2 pathway to alleviate Aβo neurotoxicity [360], casts a more favorable light upon PrP^C^ LLPS-induced Aβo binding and cascading signaling effects, further highlighting the important role of melatonin in supporting PrP^C^ physiological activities while ameliorating pathological consequences. In 2021, König et al. reported solid-state MAS NMR spectroscopy investigations of Aβ(1–42) oligomers complexed with huPrP represented a heterogeneous mixture of β-strand-rich assemblies where binding with PrP^C^ effectively trapped Aβ oligomers and prevented further development into various fibril types, prompting the authors to speculate whether this feature was coincidental or is the original intended physiological function of PrP^C^ [361]. Additional discoveries from recent experimental studies continue to deepen understanding of the complex relationship between prion LLPS, copper, the TME, and melatonin.

#### 2.3.2. Is the N-1 Fragment from the Intrinsically Disordered N-1 Domain Necessary and Sufficient for LLPS?

The mature PrP^C^ comprise two well-differentiated domains—a structured C-terminal (residues 121–231) and the unstructured N-terminal domain (residues 23–120) [333,362,363]. Within the N-terminal domain at residues 51–90, there are four octarepeats [364,365], which contain copper-binding histidines and a “pseudorepeat” lacking a histidine [333,363]. A fifth consensus copper-binding site can be found between residues 91 and 111 [363], which is an area known for amyloidogenic β-sheet formation coordinated by copper binding to His^96^ and His^111^ which results in structural plasticity changes involving “closed” or “open” conformations that are prion-resistant or prion-susceptible, respectively [366,367] (Figure 1). In addition to binding copper and other divalent metals such as nickel(II), zinc(II), and manganese(II), albeit with much lowered affinity [368], histidines in PrP and especially within the octarepeat peptides bind hemin where affinity increased with the number of histidines and length of the peptide [369]. The binding of copper to the four octarepeat peptides induces conformational changes that rapidly dissociate PrP^C^ from lipid-rafts, laterally translocating the protein from detergent-resistant lipid rafts into detergent-soluble regions of plasma membranes before endocytosis through clathrin-coated pits or caveolae [326,370,371,372,373,374]. The region containing octarepeat peptides can also be dissociated from the membrane-bound prion protein via proteolytic or ROS-induced cleavage [375,376].

The constitutive, irreversible, posttranslational proteolytic α-cleavage of residues 110/111 in PrP^C^ yields N1 (residues 23–110) and C1 (residues 111–230) fragments while ROS-induced β-cleavage at residues 89/90 produces N2 (residues 23–89) and C2 (residues 90–230) fragments [120,377,378,379,380] (Figure 1). The soluble N-terminal cleavage fragments become untethered while the C-terminal cleavage fragments remain bound to lipid rafts on membranes via GPI anchors [272,380,381]. Shedding by proteolysis releases a full-length, soluble PrP^C^ (23–230) that is cleaved from the membrane GPI anchor around reside 230–231 and reduces the cytotoxicity of amyloid-β oligomers [379,382,383] (Figure 1). The nature and function of proteolytic cleavage of PrP^C^ and perhaps even PrP^Sc^ [384,385] are yet to be fully elucidated, although it is postulated that proteolytic processing generates bioactive soluble prion protein fragments and induces conformational and functional changes to cell-bound prions [375,379,386].

Substantial evidence from experimental studies have shown that β-cleavage can also be induced by exposure to ROS (H_2_O_2_) but is dependent upon pH and Cu^2+^. Binding to copper facilitated β-cleavage by H_2_O_2_ in the octapeptide repeat region but the rate of cleavage was reduced when pH was lowered [376,387]. A higher level of α-cleavage in full-length PrP^C^ producing more C1 fragments was correlated with better resistance to the propagation of PrP^res^ [388], whereas deleting α-cleavage sites while retaining residues 23–31 produced toxic, lethal phenotypes [389]. However, biologically active N2 fragments (residues 23–89) from β-cleavage modulates cellular stress response [285], and deleting the octarepeat regions not only abolished ROS-mediated β-cleavage but also greatly reduced cell viability and increased intracellular free radicals from impaired glutathione peroxidase activity [390]. Both N1 and N2 cleavage fragments can also maintain neuronal stem cell quiescence by modulating ROS levels [391]. Cancer cell stemness contributes to MDR, and the ability to maintain stem cell pools in a quiescent, slow-growing state facilitates protection from antiproliferative drugs and evasion from immune surveillance to promote tumor development [392,393,394].

In February 2021, Tange et al. reported that at neutral pH 7.0, interactions between kosmotropic anions and N2 residues 23–89 in the N-terminal region of rPrP were most optimal in driving rPrP LLPS, forming gels that acquired conformational conversion into PK-resistant β-sheet–rich, non-seeding structures without the use of kinetic energy or PrP^Sc^ [121]. These findings support results from early experimental studies where kosmotropic anions promoted the conversion of rPrP into PrP^Sc^-like aggregates [228]. However, Kamps et al. published their report later in 2021 showing that at physiological pH 7.4, N1, but not N2, underwent LLPS driven primarily by the polybasic motif in the postoctarepeat region containing an amyloid β-binding domain [266]. Interestingly, during their experiments, Tange et al. found the presence of copper inhibited LLPS [121], whereas Kamps et al. did not test the effect of copper on LLPS [266]. Even though copper binding to histidines in PrP^C^ can induce conformational changes that could reduce potential toxicity effected by N-terminal with octarepeat sequences [294,300,377,395] but also impede LLPS, it is not inconceivable that the difference in pH of mediums used in the two studies in addition to the absence of copper and kosmotropic anions may offer a plausible explanation for LLPS observed in N2 [121,266].

#### 2.3.3. Changing pH and/or Crossing Isoelectric Points Can Drive Phase Separation of Prion N2 Fragments

Under normal physiological conditions, the pH of the human body is maintained in a tight range between 7.35 and 7.45, with 7.40 accepted as the average physiological pH [396]. Changes in pH in an organism is a critical stress factor that can induce the formation of MLOs through LLPS [124,397,398]. Results from in vitro experimental studies demonstrate that changes in pH can trigger phase separation of stress sensing poly(A)-binding proteins in yeast to form hydrogels [190]. Under nutrient depletion, yeast cells are unable to regulate pH using proton pumps; the ensuing acidification triggers phase separation, reversibly transitioning the yeast cytoplasm from a fluid- to a solid-like, dormant state with reduced mobility [399]. Prions can undergo huge conformational changes below pH 7.2 when interacting with nucleic acids, forming large RNA–protein complexes in a pH-dependent manner [400,401], whereas increasing concentrations of chaotropic salts such as sodium chloride (NaCl) at pH 7.5 prevented the formation of RNA prion complexes [400,402]. Reducing pH can cause thermodynamic instability propelling the conversion of PrP^C^ into PK-resistant isoforms by destabilization of salt bridges in nucleic acids and protonation of histidine residues in PrP^C^ [403,404]. Conversely, increasing pH can cause histidine residues that serve as molecular switches in histidine-rich squid beak proteins (HBPs) to deprotonate and trigger phase separation [405]. pH jumps from pH 11.0 to pH 7.5 caused proteins kept in solution to quickly undergo LLPS to form droplets upon protonation at native pH. Decreasing pH is often used as an effective technique to induce LLPS in proteins without having to cross the isoelectric point of the proteins [406].

Phase separation can often be triggered as the pH moves close to a protein’s isoelectric point (pI), which is the pH value at which a molecule carries no net electrical charge where the negative and positive charges are equal or cancelled. Therefore, proteins will carry a net positive charge if the pH of the surrounding liquid medium is below their pI and a net negative charge if the surrounding pH is above their pI [407]. Experimental results indicate that phase separation frequently occurs at pH values corresponding to the protein’s isoelectric point at thermodynamic equilibrium, whereas cells are almost always under nonequilibrium conditions that may also affect phase separation [408]. Nonetheless, proteins were shown to be the least soluble near their pIs where solubility is affected by the increase in net charge, which may be proportional to increases or reductions in the surrounding pH [409], with the implication that a net charge of zero may induce protein aggregation. Testing of several disease-associated transmissible spongiform encephalopathies (TSEs) human prion proteins (PrP^TSE^) found their isoelectric points to be more acidic than pH 7 [410], which may explain why Tange et al. observed LLPS of N2 fragments at neutral pH in the presence of kosmotropic anions, and Kamps et al. were unable to induce LLPS of N2, which lacked the postoctarepeat region with the amyloid β-binding domain, at physiological pH 7.4 [121,266]. Copper-binding, which can interfere with LLPS, is also pH dependent.

#### 2.3.4. Copper Chelation by Melatonin in Prion Phase Separation May Ameliorate Prion-Induced Multidrug Resistance

At neutral or physiological pH copper (Cu^2+^) is fully bound to histidine residues in the octarepeat and other regions of PrP^C^ at a 1:1 ratio [411,412]. Reducing pH to 6.7 results in loss of binding by 50%, and further reductions to pH 6.0 completely inhibited binding [93], or led to dissociation of the Cu(II)-amide^−^ bonds [411]. Although normally found bound to proteins, Cu(II) may be released and become free to catalyze the formation of highly reactive hydroxyl radicals inducing cellular toxicity [413,414]. Exchangeable copper (CuEXC) represents the labile fraction of copper complexed to albumin and other peptides but not within ceruloplasmin [415,416]. In the healthy individuals tested, CuEXC was found to be 0.57 to 1.12 μM, or 3.24% to 8.58% of total copper concentration in plasma [417], which is normally ~16.7 μM on average [418], whereas human and murine prions are almost fully saturated at 5 μM copper [419]. Copper is increasingly associated with the growth and proliferation of cancer cells and the promotion of breast cancer metastasis [420,421]. Thus, in environments below neutral pH—the hallmark of cancer TME—prions may not bind to copper completely, which then becomes a challenging situation in the context of cancer MDR.

Prions are copper-sensitive stress sensors that are activated upon copper-binding to initiate signal transduction processes that increase antioxidant enzyme activities and glutathione levels [19,422]. Exposure to Cu(II) was shown to increase the expression of PrP^C^ in primary hippocampal and cortical neurons [423], and increased oxidative stress induced by intracellular Cu(II) quickly upregulated PrP^C^ transcription mediated by ataxia-telangiectasia mutated (ATM) in murine neuro-2a and human HeLa cells [424]. In addition, ROS-mediated β-cleavage at residues 89/90, which produces N2 (residues 23–89) [379,425], is also copper- and pH-dependent, with the rate of cleavage at neutral pH diminishing with decreasing pH [376]. PrP mutants lacking the copper-binding octarepeat peptides could not undergo β-cleavage by ROS and displayed increased sensitivity to oxidative stress [390]. Hence, in an acidic extracellular environment favored by cancer cells, prion expression may be elevated due to increased oxidative stress from incomplete Cu(II) binding, which also results in suppressed antioxidant protection from copper-dependent ROS-mediated β-cleavage [390]. Increased oxidative stress and a lower pH will also trigger PrP^C^ phase separation, which may lead to the aggregation of the pathological PK-resistant isoforms. Oxidative stress causes prion protein misfolding and a 900-fold increase in binding affinity, resulting in oligomerization that seeds aggregation [300]. In the aggregated, PK-resistant pathological state, the prion isoform can potentially facilitate non-Mendelian, epigenetic inheritance, which confers stress and drug-resistant survival features to cancer cells [40,426].

Melatonin is not only a highly efficient antioxidant that continues to generate effective free radical scavenging metabolites while interacting with different ROS [126,127,128,129,130,131,132,133,134,135], but also binds with copper in situ [427] and may exert protective effects against copper-induced toxicity in animals and plants potentially via chelation [428,429]. Under physiological conditions, in vitro and in vivo animal experiments found melatonin treatment at 1 mM and 50 mg/kg (intraperitoneal injection), respectively, decreased hydroxyl radical formation by high concentration of copper and pro-oxidant polyphenols, preventing DNA damage via copper chelation [430]. A theoretical study employing physicochemical analysis in 2015 proposed that under physiological pH 7.4, melatonin can chelate Cu(II) via the coupled-deprotonation-chelation mechanism (CDCM), with 3-hydroxymelatonin (3OHM) being the most effective metabolite for such purpose [431]. In 2019, computational studies simulating physiological mediums reported results that supported these findings. However, when comparing Gibbs free energies between melatonin complexes formed with various metals examined using the well-known metal-chelating agent ethylenediaminetetraacetic acid (EDTA) [432] as control, copper complexed with melatonin and principal metabolites showed the lowest Gibbs free energy values in the order of EDTA, AMK, 3OHM, melatonin, and AFMK, where EDTA- and AMK-Cu complexes exhibited the highest stabilities with the lowest Gibbs free energy at approximately −161 and −149, respectively [433].

**Figure 2:**
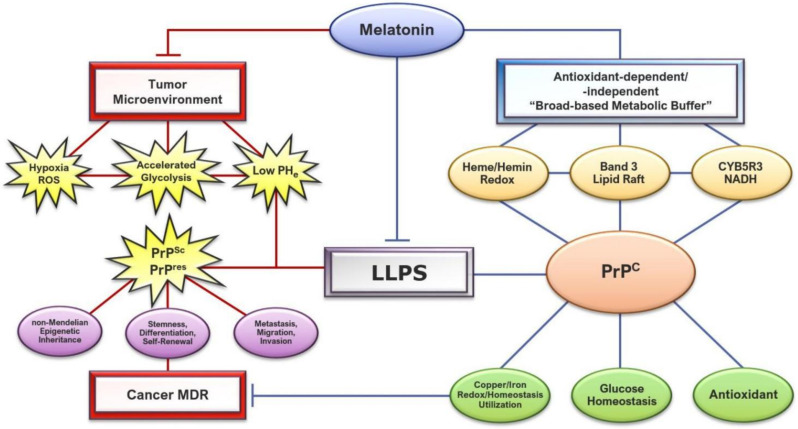
Schematic illustrating the regulation of prion protein phase separation by melatonin, attenuating conversions into infections, pathological PrP^Sc^ and non-infectious, insoluble PrP^res^ isoforms, which may promote cancer multidrug resistance (MDR) via different mechanisms, including non-Mendelian epigenetic inheritance, stemness, metastasis, and resistance to apoptosis. By acting as a “broad-based metabolic buffer”, melatonin modulates the tumor microenvironment to control hypoxia and oxidative stress, ameliorating the effects of accelerated glycolysis and low extracellular pH (pH_e_) that can trigger the liquid–liquid phase separation (LLPS) of physiological prion (PrP^C^). Melatonin employs antioxidant-dependent and -independent features to protect heme redox and NADH levels; band 3, lipid raft, and CYB5R3 functionality; and, provides an optimal environment for prions to assume essential physiological functions including reduction in oxidative stress, maintenance of cellular energy homeostasis, and ensuring proper iron/copper redox/homeostasis and utilization, which may further enhance cancer drug sensitivity.

Considering the fact that deprotonation increases the chelation viability for Cu(II), reduced pH can, therefore, negatively impact melatonin’s ability to chelate copper [431]. However, it is perhaps not a coincidence that melatonin increases pH, restoring pH homeostasis to regulate prion phase separation, facilitate copper-binding, and modulate ROS-mediated cleavage via a reduction in oxidative stress through its potent antioxidant cascades [434]. Treating irradiated healthy and tumor-control Balb/c mice with melatonin (20 mg/kg) ameliorated oxidative stress in heart and lung tissues. However, melatonin administration increased superoxide dismutase (SOD) and glutathione peroxidase (GPx) antioxidant responses only in normal but not tumor cells [435]. It is plausible that by reducing ROS levels in oxidative TMEs, melatonin decreased PrP^C^ expression, which in turn lowered antioxidant activities. Results from an in silico analysis demonstrated that the overexpression of PrP^C^ under optimal culture conditions did not alter proliferation, resistance to cell death, and metabolism in colorectal cancer cell lines [436], and consequently, supported the hypothesis that the correlation between overexpression of PrP^C^, cancer malignancy, and MDR are actually results of a highly-stressed TME rather than outcomes being driven by PrP^C^ overexpression. The ability of melatonin to act as a “broad-based metabolic buffer” which can tune prion propagation in response to stress signals becomes particularly significant in the context of TME and drug resistance (Figure 2).

## 3. Melatonin May Promote PrP Physiological Functions and Inhibit Pathological Effects via Global Modulation of the Tumor Microenvironment to Enhance Cancer Drug Efficacy

One of the major metabolic adaptations employed by cancer cells is the “Warburg effect” where mitochondrial oxidative phosphorylation (OXPHOS) is suppressed in favor of accelerated aerobic glycolysis [437], producing a toxic tumor microenvironment (TME) characterized by high alkalinity in the cytosol and high acidity in the extracellular environment resulting in an elevated alkaline intracellular pH (pH_i_) but an acidic, reduced extracellular pH (pH_e_) that can promote oncogenic properties [438,439]. This reversed pH gradient is widely accepted as the hallmark of cancers [440,441]. Cancer cells have been associated with higher values of pH_i_ between 7.12 and 7.65 and a lower pH_e_ of ~6.2–6.9, whereas pH_i_ in normal cells is stringently maintained at a narrow range between 7.0 and 7.2, and pH_e_ at ~7.4 [442,443,444,445,446,447,448,449]. In normal cells, metabolic and developmental transitions are highly dependent upon changes in pH_i_ [450,451,452] and in silico studies showed that alkaline pH_i_, which is coupled to accelerated glycolysis and adaptation to hypoxia, maximized cancer cell proliferation, whereas reversing the pH_i_ to normal acidic values prevented adaptations, halting tumor cell growth [453]. An acidic pH_e_ in the TME is directly correlated to deficient oxygen supply from rapid cancer cell division and growth.

Tumor hypoxia causes the metabolic shift towards acidity where proton (H^+^) accumulation is proportional to O_2_ levels [454]. Excess intracellular protons are often extruded into extracellular space via different mechanisms [455] including membrane transporters [456], carbonic anhydrase enzymes [457], and lysosomes [458], or sequestered in proton sinks [459]. The ensuing acidic pH_e_ may directly interfere with the efficacy of weakly basic chemotherapeutic drugs by impeding their intracellular distribution through “ion trapping” [460]. While the combination of proton disequilibrium and reversed pH gradient act as positive feedback promoting metastasis that exacerbate cancer MDR [441,447,461,462,463], it is the fall in intracellular proton that is mainly responsible for accelerated glycolysis in cancer cells [464]. Since mitochondria ATP synthases are rapidly translocated to cell surface lipid rafts under tumor-like hypoxic and acidic environments [465,466,467], cancer cells can also rely on the internalization of extracellular ATP (eATP) to significantly elevate intracellular ATP (iATP) to enhance drug resistance by maintaining the energy requirement of drug efflux by ATP-binding cassette (ABC) transporters [468,469,470]. eATP has been associated with cancer cell migration and invasion [471,472], induction of epithelial-mesenchymal transition (EMT) to promote metastasis in lung cancer [473], and activation of cancer stem cell-like changes to promote metastasis in non-small-cell lung cancer [474].

### 3.1. Melatonin May Attenuate Prion Propagation and Cancer Multidrug Resistance by Increasing Extracellular pH

Extracellular acidification and hypoxia in melanoma cells can reprogram metabolism to enhance survival, invasiveness, and promote immunosuppressive environments that exacerbate drug resistance [475]. Hypoxia induces increased expression of cellular prion protein to enhance the viability of mesenchymal stem cells [476], and PrP^C^ mRNA and protein levels were significantly upregulated (4.3-fold increase in luciferase activity) in gastric cancer cell lines exposed to hypoxia [59]. In fact, increased expression of PrP^C^ in multicellular prostate tumor spheroids is regulated by redox to counterbalance increased oxidative stress through upregulated antioxidant defense [477]. Prion phase separation can be activated by cellular stress such as changes in pH and fluctuations in levels of kosmotropic anions including ATP [121,226,406,455] (see Section 2.2.1.). Biopsies from metastatic melanoma revealed elevated levels of amyloid-like aggregations [478], and amyloidogenic peptides were shown to incorporate ATP when aggregating into amyloid fibrils [479]. Increased eATP in addition to increased oxidative stress and reduced pH_e_ in TME may exacerbate prion β-sheet conversions upon triggering of phase separation. Even though LLPS converted rPrP into the PK-resistant PrP^res^ isoform, it is still unclear whether phase separation of PrP^C^ is the primary cause for the conversion of PrP^C^ into PrP^Sc^. However, oxidative stress is increasingly associated with the conformational change in the α-helix structure of PrP^C^ to the β-sheet structure of PrP^Sc^ [480,481,482,483]. It is not surprising that the migration of metastatic melanoma, which is dependent on acidic pH_e_, is promoted by the prion protein [67,484,485]. Experimental studies on skin reconstructed with melanoma cell lines found treatment with 1 mM melatonin controlled growth and impaired invasion and metastasis by disrupting cytoskeleton formation [486] while high-dose melatonin (5 mg/m^2^/day to 700 mg/m^2^/day) showed stable, favorable responses in human subjects diagnosed with advanced malignant melanoma [487]. Melatonin also prevented the aggressive phenotype shifts in breast cancer cell lines maintained under acidosis conditions by modulating proliferation and apoptosis [488]. Melatonin can exert inhibitory oncostatic effects due to its ability to regulate acid-base balance fluctuations, which are consequences of a hypoxic TME [454].

In vitro experimental studies showed that exposure of two human pancreatic cancer cell lines (MIA PaCa-2 and PANC-1) to 1 μM melatonin with continuous presence (including measurement) for 24 h stimulated the secretion of bicarbonate, rebalancing ion transport via modulating mRNA expression of pancreatic solute transporters SLC26A6, SLC4A4b, SLC9A1, and other non-genomic effects on acid-base transport that were not identified [489]. Expressed in all cells, carbonic anhydrases (CAs) are catalytic enzymes responsible for the reversible conversion of carbon dioxide (CO_2_) and water (H_2_O) into bicarbonate (HCO_3_^−^) and protons (H^+^) [490,491]. The CA isoforms CA-IX and CA-XII contribute to extracellular acidification and intracellular alkalinization in response to increased CO_2_ load under hypoxic conditions. The reversed pH gradient of increased pH_i_ and reduced pH_e_ is a major pro-survival mechanism used by cancer cells [492,493]. In vivo experiments showed that silencing of CA-IX led to a 40% reduction in xenograft tumor volume with up-regulation of CA-XII levels, whereas invalidation of both isoforms produced an impressive 85% reduction [494].

Melatonin treatment of triple negative human breast cancer cell line (MDA-MB-231) and female Balb/c xenograft mice at 1 mM and 40 mg/kg, respectively, showed slightly different results between gene expression and protein levels of CAs. Tumor samples from xenograft mice treated with high-dose melatonin exhibited significant downregulation of mRNA gene expression of CA-XII and markedly reduced protein levels of both CA-IX and CA-XII when compared to untreated controls, whereas in vitro results from cultured MDA-MB-231 cancer cells treated with 1 mM melatonin only showed a significant reduction in CA-XII gene expression, with an insignificant difference in protein levels of CA-IX and CA-XII between the melatonin-treated and control groups [495]. Since CA-IX and CA-XII are inducible by hypoxia, in the same study, melatonin also reduced gene expression and protein levels of hypoxia-inducible factor 1α (HIF-1α) in vitro and in vivo [495,496]. Reversed pH gradients with dysregulated acid-base balance in TME may be consequences of hypoxia where arterial hemoglobin desaturation and reduced O_2_ saturation can lower pH_e_ to below 6.8 [497,498]. Low partial pressure of oxygen (pO_2_) can directly affect resistance to radiotherapy by limiting the ability of O_2_ to general free radicals to exert oxidative damage to macromolecules and membranes [447,499]. Reducing the affinity of hemoglobin for oxygen represented by a right-shift in the hemoglobin-oxygen dissociation curve [500,501] can drastically increase tumor radiosensitivity [502], whereas tumor hypoxia, by lowering pO_2_ which shifts the dissociation curve to the left, thereby increasing hemoglobin affinity to O_2_, is often associated with less effective radiation-mediated apoptosis and increased metastatic potential with poorer prognosis [503,504]. The fact that melatonin exerted higher efficacy in modulating pH in vivo may reflect the powerful, dynamic relationship with prions in the regulation of iron homeostasis and hemoglobin O_2_ saturation, which control hypoxia and the resulting pH imbalances that exacerbate cancer proliferation and MDR.

### 3.2. PrP^C^ Protective Physiological Responses and Ligand-Binding May Become Pathological Liabilities in the Tumor Microenvironment

The tumor environment is uniquely adapted to promote cancer cell survival and proliferation. Elevated hypoxia from low oxygen tension produces low pH with increasing accumulation of protons (H^+^) resulting in the formation of excess ROS [454,505] and deficient energy supply are all high-stress conditions that may trigger phase separation survival responses [258,261,506] with potential to activate PrP^C^ conversion to pathological templates that may promote cytoplasmic inheritance to increase survival rates [39,196,199,200]. PrP^C^ was identified in the nucleus of NB4 human promyelocytic leukemia cell line [507], and also in the form of ‘granules’ in nuclei of uninfected bovine neuronal cells [508]. The fact that PrP^C^ is abundantly localized in the nuclear lamina and interacts with structural chromatin components [509] supports the hypothesis of PrP epigenetic regulation where prions can facilitate inheritance of activated chromatin states to provide adaptive advantages [40,41]. PrP^C^ identified in the nucleus of actively dividing normal epithelial cells was associated with the proliferation, differentiation, and subcellular distribution of architectural proteins [510]. In *S. cerevisiae*, prion-forming protein Sup35 PrD phase behavior is modulated by stress and energy depletion where droplet formation under arsenite stress and energy depletion was observed in 93% of cells examined [251]. The identification of LLPS in the nucleus further emphasizes the important role of PrP^C^ conversion from stress-induced phase separation resulting in tumor cell genomic instability [511] and dysregulation of gene expressions [259].

Most of the physiological functions of PrP are dependent on complex interactions with its binding partners. The unstructured N-terminal domain between residues 23 and 120 contains an octapeptide repeat region (residues 51–90) and an amyloidogenic region between residues 90 and 120 involving histidines 96 and 111, which bind metals with a special high affinity for copper [272,294,367,412,512], while residues 23–90 of the unstructured N-terminal constitute a region that specifically targets to lipid rafts, and PrP with deleted N-terminal is unable to bind to lipid rafts [325]. The constitutive, tight association between PrP^C^ and lipid rafts [326,513] and its wide expression in stem cells [8,514,515,516,517] offer additional insight as to how prions interact with membrane supramolecular complexes [518] to participate in an extensive range of physiological functions including transcription, scaffolding, and signaling [267], and modulate cancer stemness, differentiation, self-renewal, and proliferation to augment cancer MDR [65,71,77,516,519,520]. Although PrP^C^ does not bind iron directly, the binding of Cu^2+^ in the N-terminal domain modulates iron metabolism through copper homeostasis [289]. Wild-type (WT) PrP^C^ over-expression or deletion in specific mouse brain regions is associated with striking variations in levels of copper, iron, and even zinc [521]. PrP^null^ mice showed reduced iron mobilization, diminished serum iron content, and excess accumulation in liver and spleen as a result of impaired copper-dependent ceruloplasmin (ferroxidase) activity, which is responsible for the regulation of iron mobilization [522,523].

### 3.3. Interactions between PrP^C^, Iron, and Heme May Enhance Aggressive Drug Resistance in Tumors

Iron is required in essential metabolic processes [524], and PrP may perform important roles in iron uptake and transport [22]. Absence of PrP induces systemic iron deficiency in PrP^KO^ mice caused by less efficient uptake by red blood cells (RBCs), liver, and brain as the result of impaired transport of iron from the duodenal enterocytes—a condition that can be easily reversed by expressing WT PrP [23]. Similarly, over-expression of PrP^C^ increased intracellular iron, cellular labile iron pool, and iron content of ferritin leading to a decrease in total cellular content of transferrin (Tf) and transferrin receptor (TfR) proteins responsible for iron uptake, but an increase in ferritin responsible for iron storage [525]. Iron dyshomeostasis in brain neurons may be caused by sequestration of iron by the insoluble, aggregation-prone, infectious PrP^Sc^ isoform, which can form complexes with ferritin to induce bio-insufficiency [526]. Dysregulated iron homeostasis in cancer energy metabolism may be an important contributing factor in cancer drug resistance.

Aerobic glycolysis, commonly referred to as the “Warburg effect” [527], is undoubtedly the hallmark of cancer cells [437,528]. Enhanced, accelerated aerobic glycolysis has been shown to be responsible for resistance against various cancer drugs including sorafenib [529], palbociclib [530], oxaliplatin [531], doxorubicin [532], lapatinib [533] paclitaxel [534], bevacizumab [535], and cetuximab [536]. However, recent studies also revealed that many cancers such as myeloid leukemia [537], non-Hodgkin’s lymphoma [538], pancreatic ductal adenocarcinoma [539], melanoma [540], and high-grade prostate cancers [541] do not have impaired mitochondrial OXPHOS [542] while aggressive and drug-resistant cancers may actually upregulate mitochondrial oxidative phosphorylation (OXPHOS) as part of their defense mechanisms [543,544,545] to enhance autophagy [546], increase stemness [547], or remodel OXPHOS metabolism to promote survival [541,548].

Under physiological conditions, ATP hydrolysis is tightly regulated and the standard energy (ΔG′_ATP_) is maintained between 53 and 60 kJ/mol, where 56 kJ/mole, in principle, is regarded as the endpoint of both genetic and metabolic processes required for sustaining life [549,550]. Chemical energy of ATP is primarily used to power ionic membrane pumps that support cell and organ viability [551]. Uncontrolled proliferation, heightened dedifferentiation, and resistance to apoptosis in cancer cells may be the result of survival mechanisms activated in response to chemical energy deficiencies [549,552]. The exploitation of iron-containing heme is a preferred and highly effective counter-strategy employed by cancer cells to modulate energy metabolism and reprogram their environment [553,554,555]. Iron metabolism is vital for normal and cancerous cells [524,556]. The regulation of iron homeostasis in carcinogenic mechanisms has been extensively discussed and reviewed [554,556,557], where targeting iron metabolism via iron depletion or iron overload is considered a formidable anti-cancer strategy [558,559]. In addition, large cohort studies have also discovered a positive correlation between dietary heme iron intake and colon carcinogenesis [560,561].

#### 3.3.1. Iron and Heme Facilitate Increased Energy Production in Cancer Cells

Iron is a transition metal with essential physiological functions including oxygen transport and production of cellular energy [524]. However, the two primary biological redox states of Fe^2+^ and Fe^3+^ can also catalyze the generation of hydroxyl radicals (^•^OH) through the Fenton reaction [562]. The pleiotropic relationship between iron and oxygen began ~3.5 billion years ago when cyanobacteria first introduced oxygen (O_2_) to earth’s water and atmosphere via water oxidation in the production of ATP [563,564,565,566]. During mitochondrial OXPHOS, oxygen consumption by cytochrome c oxidase (COX or complex IV) may reach 90% of total cellular oxygen [567] as part of the O_2_ reduction process that maintains the proton-motive gradient via proton pumping across the inner mitochondrial membrane. Proton pumping is mainly powered by the creation of a net positive charge via the oxidation of low-spin heme iron in COX [568,569]. In the human body, most of the iron is contained in heme proteins such as hemoglobin, myoglobin, and cytochromes [570,571]. The important, terminal step that completes the biosynthesis of heme occurs on the inner surface of the inner mitochondrial membrane (IMM) where ferrous iron (Fe^2+^) is inserted into the tetrapyrrole macrocycle of protoporphyrin IX (PPIX) by ferrochelatase [571,572,573].

Mitochondrial respiration is dependent upon homeostasis of the heme synthesis-export system, which regulates the tricarboxylic acid cycle (TCA) and controls the rate of OXPHOS where reduced heme synthesis or hypoxia induces heme export to shut down OXPHOS and activates glycolysis. However, the feedback effect of heme-export in turn increases heme synthesis, which can fuel increased TCA-cycle flux and OXPHOS rates [574]. Breast and lung cancer cells exhibit abnormal upregulation of the feline leukemia virus subgroup C receptor 1 (FLVCR1) heme-exporter [575]. Inhibition of FLVCR1 in breast and lung cancer cells resulted in dramatic reductions in proliferation, migration, invasion but acceleration in apoptosis [576,577,578]. Vascular disrupting agents (VDAs) such as combretastatin A-4 phosphate (CA4P) that are used to treat solid tumors often result in increased tumor recurrence and post-VDA treatment resistance because even though VDAs reduce tumor oxygenation, they also trigger upregulated heme flux, biosynthesis, uptake, and degradation [579] as defense mechanisms. Enhanced heme function leading to increased mitochondrial energy production fueling proliferation and progression is a classic feature of aggressive, high-mortality non-small-cell lung cancers (NSCLCs) [580] and other chemoresistant cancers [581]. PrP binds to both heme and hemin in human RBCs.

#### 3.3.2. PrP^C^ Regulates Heme Synthesis and Export to Modulate Glucose and Antioxidant Homeostasis in Cancer

PrP^C^ is widely expressed in human blood where the number of prion molecules bound per blood cell was detected to be 290 ± 140 on red blood cells [582], 619 ± 167 on platelets, and 11,363 ± 2320 on lymphocytes [583]. Since the normal number of RBCs in man is ~5 × 10^9^/mL, it is reasonable to assume that RBCs may be the main source of cell-associated PrP^C^ in human blood [582]. Each of the four iron PPIX–heme complexes within hemoglobin of RBC contains an iron ion existing in either the reduced ferrous (Fe^2+^) state in heme, or the oxidized ferric (Fe^3+^) state in hemin [584,585,586,587]. PrP is a physiological ligand of both heme and hemin, and may be responsible for regulating heme homeostasis and heme redox activities. The in vitro direct interaction between heme (Fe^2+^) and PrP^C^ not only enhanced peroxidase activity, but also inhibited the conversion of PrP^C^ to PrP^Sc^ while preventing fibril formation in the heme-amyloid-β complexes [588]. By contrast, hemin is the PPIX–heme complex with iron in the oxidized ferric (Fe^3+^) state and can generate ROS through the Fenton reaction [562]. The prion protein exhibits great affinity for hemin, and binding to hemin causes PrP to form insoluble aggregates in vitro; yet hemin (Fe^3+^) bound to PrP^C^ also exhibited enhanced peroxidase activities with the implication that PrP^C^ possesses inherent protective, antioxidant functions [369,589]. In fact, brain lysates from PrP knockout mice had higher levels of oxidative damage to proteins and lipids compared to WT mice of the same genetic background [590]. In addition, cultures of primary cerebellar granule neurons derived from PrP knockout mice were highly susceptible to H_2_O_2_-induced toxicity as a result of significantly decreased glutathione reductase activities measured in vitro and in vivo [591].

Drug resistant cancers often display increased antioxidant defense via upregulation of reduced glutathione (GSH) production through metabolic modulation favoring a glycolytic shift that activates the pentose phosphate pathway (PPP) [592]. Recent evidence showed that both the glucose-6-phosphate dehydrogenase (G6PD) pathway and a less characterized hexose-6-phosphate dehydrogenase (H6PD) pathway contribute to accelerated cancer cell growth [593]. In breast cancer, hyperglycemia is an important factor that can reduce chemotherapy efficacy by promoting proliferation, invasion, migration, and anti-apoptotic defenses via accelerated glucose metabolism [594]. Breast cancer MCF-7 cell lines resistant to adriamycin showed increased glucose metabolism with heightened expression of glucose transporter GLUT1 [595,596]. An important physiological function of PrP^C^ is the maintenance of glucose homeostasis through regulation of intracellular iron levels that control glucose metabolism through heme synthesis [597]. Pancreatic iron stores in PrP knockout mice were significantly lower than WT controls and silencing expression of PrP^C^ in human pancreatic β-cells (1.1B4) significantly lowered intracellular iron and dramatically upregulated GLUT1 and GLUT2. By contrast, iron overloading downregulated glucose transporters GLUT1 and GLUT2 in a PrP^C^-dependent manner [15]. Experimental results showed that PrP^C^ may act as an ancillary protein that is required for the function and expression of GLUT1 where PrP^C^ depletion inhibited glucose utilization in human colorectal carcinoma cell lines and a human colorectal xenograft model in nude mice, with significant reductions in proliferation and survival of cancer cells both in vitro and in vivo [598]. In addition, prion-like aggregates of the islet amyloid polypeptide (IAPP) in the islets of Langerhans were proposed to play important roles in causing β-cell dysfunction and loss resulting in insulin resistance and hyperglycemia [599]. IAPP binds to heme-forming complexes, which facilitates the production of partially reduced oxygen species (PROS) that can damage β-cells [600,601,602,603].

Heme controls glucose regulation via direct interactions with insulin at two high-affinity insulin heme-binding sites, and heme-insulin complexes exhibit enhanced peroxidase activity and increased insulin cross-linking that lead to permanent loss of insulin functionality [604]. Increased heme levels and export from elevated FLVCR1 mRNA expression in adipose tissues of T2D patients were positively correlated with fasting glucose, triglycerides, and serum ferritin; but negatively correlated with insulin sensitivity [605]. The binding of hemin to prion may be a protective, physiological response that defends heme homeostasis since hemin with oxidized, ferric iron is unable to bind oxygen [606]. Hemin is potentially cytotoxic [607,608,609] due to its ability to inhibit glutathione S-transferase activity through competitive binding in human erythrocytes [610] and cause degradation and covalent cross-linking of glutathione reductase in yeast models [611]. Hemin bound to PrP^C^ exhibits increased peroxidase activity compared to free hemin as a result of the coordination of PrP^C^ octarepeat peptide region residues 34–94 to ferric iron in hemin [284,589]. However, this initial increase in peroxidase activity over a longer time frame may eventually elevate oxidative stress causing aggregation of insoluble PrP^C^ isoforms [369,483] which can potentially change the conformation and physiological functions of PrP^C^. In cancer cells, heme serves important functions in the regulation of cell cycle and cell growth. Inhibition of heme synthesis caused cell cycle arrest, senescence, and apoptosis [612]. Therefore, increased oxidative stress in the TME [613] may elevate prion-hemin binding, resulting in increased tumor MDR.

#### 3.3.3. Upregulation of Hemoglobin Synthesis by Hemin-Bound PrP^C^ May Increase Cancer Multidrug Resistance

The binding of hemin to PrP^C^ in diverse cell lines results in aggregation or degradation of PrP^C^ in a cell-type specific manner. However, the binding interaction also significantly upregulates hemoglobin synthesis in hematopoietic cells, where brain organotype cultures exposed to hemin showed increased α-globin in PrP WT compared to PrP knockout samples. Additionally, RBCs from PrP knockout mice had markedly lower α-globin levels compared to PrP WT controls [614]. Since heme regulates gene expression transcriptionally and post-transcriptionally [615,616], heme can initiate changes in key factors that control extensive processes from cell cycle and Ras signaling to chromatin structure, splicing, and protein folding [617,618]. Heme controls chromatin and genome function previously not associated with heme regulation [619]. Thus, the upregulation of heme synthesis as a result of PrP^C^ binding to hemin may be a significant factor contributing to cancer drug resistance [553]. Even though PrP^C^ bound to hemin (ferric PPIX) showed rapid precipitation with increased aggregation and decreased solubility [369,589], in vitro heme (ferrous PPIX) interaction with PrP^C^ inhibited the seeded conversion of PrP^C^ to PrP^Sc^ in protein misfolding cycling amplification assays where conversion could be inhibited at heme concentrations from 10 to 1000 μM but not at 1 μM [588]. Porphyrin tetrapyrroles (IC_50_ ~0.5–1 mM) inhibited the formation of PK-resistant PrP without affecting the biosynthesis of normal PK-sensitive PrP in scrapie-infected mouse neuroblastoma (ScNB) cell cultures [620]. If the redox cycling between heme and hemin is intended as a natural feedback control for prion conversions, then the elevated ROS in TME together with increased oxidative stress from prolonged peroxidase activity from PrP^C^-hemin complexes [369,588] may terminate the feedback cycle to favor increased hemin-PrP^C^ binding that heightens cancer drug resistance as a result of elevated hemoglobin synthesis. Using melatonin to restore heme–hemin redox balance may prevent conversion of PrP^C^ to PrP^Sc^ and preserve PrP^C^ physiological functions while enhancing cancer drug efficacy.

### 3.4. Melatonin Maintains Hemoglobin Redox Balance by Protecting CYB5R3 and Band 3 Protein in an Antioxidant-Independent Manner

Due to the natural redox state of ferrous and ferric iron in heme, hemoglobin can become “biologic Fenton reagents” which readily promote hydroxyl radical formation [621]. Therefore, erythrocytes (red blood cells) must depend on robust antioxidant systems to maintain heme redox balance [622,623,624]. The physiological autoxidation of hemoglobin (0.5–3%/day) creates the reversible hemin (ferric PPIX) derivative, commonly known as methemoglobin (MetHb) [625,626], where the sixth coordination position of the heme iron is occupied by either hydroxide (OH^−^) or water (H_2_O) [627]. The water molecule coordinated to the iron atom in ferric MetHb results in increased instability compared to ferrous heme, and can also cause significant loss of heme at rates substantially higher than even ferrylHb (Fe^4+^) [628]. In addition, MetHb cannot bind oxygen and must be effectively reduced back to the ferrous state by NADH-cytochrome b5 reductase 3 (CYB5R3). CYB5R3, also known as NADH–cytochrome b5–metHb reductase, is a flavoprotein responsible for the transfer of electrons from NADH via cytochrome b5 (CYB5) to reduce MetHb, producing NAD^+^ [629,630]. CYB5R3 exists in two isoforms, where the soluble isoform is found exclusively in RBCs [631,632], and the membrane-bound isoform is ubiquitously expressed in mammalian cells including erythrocytes, mitochondria, and lipid rafts [630,633,634,635,636].

Elevated oxidative stress in the TME [613,637] may challenge antioxidant systems in RBCs leading to increased formation of MetHb and the release of free heme that can be complexed with PrP^C^. Rapid depletion of NADH in erythrocytes exposed to oxidants such as T-butylhydroperoxide resulted in elevated MetHb due to increased consumption to support recovery of reduced glutathione [638]. However, in 1999 when Tesoriere et al. exposed human erythrocytes to cumene hydroperoxide (cumOOH) to induce the oxidation of a 1% suspension of RBCs, which led to 100% hemolysis of samples in 180 min, the addition of 50 µM melatonin effectively delayed denaturing of hemoglobin and release of hemin in an antioxidant-independent manner. Melatonin treatment inhibited hemin precipitation in oxidized RBCs compared to controls where increased hemin swiftly partitioned into RBC membranes. Even though MetHb may be responsible for the generation of additional ^•^OH, and melatonin is a potent scavenger of hydroxyl radical [126] with its relatively low oxidation potential of approximately +570 mV [639] compared to ^•^OH [640], the protective effects observed by Tesoriere et al. were not related to antioxidant functions. Nevertheless, 35% of melatonin was consumed by RBCs under cumOOH challenge, while no melatonin was consumed by reactions with ^•^OH in the experiment [641]. Six years later, Tan et al. demonstrated that melatonin may have been utilized to recycle NADH to regenerate CYB5R3 in the reduction of MetHb [642].

Tan and colleagues reported for the first time in 2005 that melatonin is able to recycle NAD^+^ to NADH, forming the N1-acetyl-N2-formyl-5-methoxykynuramin (AFMK) metabolite in the process through the cleavage of the pyrrole ring [642,643]. Melatonin is an ideal electron donor due to its electron-rich aromatic indole ring [644]. The use of 1 millimolar (mM) melatonin prevented the loss of NADH in PC12 cells subjected to 150 µM paraquat incubation while 2000 µM MEL provided greatest protection to NADH loss from 500 µM orthovanadate (Va^5+^) incubation [642] (Table 1). In the absence of NADH, melatonin reduced autoxidation of human oxyhemoglobin (HbO_2_). Autoxidation was increased when HbO_2_ was incubated with NADH and the effect was profoundly augmented by the addition of melatonin (each at 500 µM). However, addition of melatonin did not change the level of NADH consumption even though HbO_2_ autoxidation was markedly elevated. Since NADH levels remained constant, it was concluded that the presence of melatonin recycled NADH through electron donation to form AFMK as metabolite [642]. Melatonin can also protect band-3 protein at the membrane level in an antioxidant-independent manner. Addition of 300 μM H_2_O_2_ to erythrocytes decreased expression of band 3 and altered cell shapes without causing lipid peroxidation or formation of MetHb. In the absence of catalase, the addition of 100 μM melatonin reversed RBC cell-shape changes and restored band 3 protein conformation and expression levels. Interestingly, treatment with 1 μM melatonin was ineffective and even caused cell-shape changes and increased lipid peroxidation in RBCs challenged with H_2_O_2_ [645,646]. The fact that melatonin at pharmacological doses exerted opposite effects on RBCs is reminiscent of various observations where low and high doses achieved opposite effects in stimulating or inhibiting prion activities, respectively [114,118] (Table 1). Regardless, the protection of band 3 by melatonin may be a significant contributing factor in the attenuation of TME-associated hypoxia and accelerated glycolysis, which directly modulate PrP^C^ phase separation and related functions.

### 3.5. Melatonin Increases O_2_ Saturation to Reduce TME Hypoxic Stress by Protecting Band 3 Protein

Hypoxia is an environmental selection pressure that can significantly exacerbate cancer drug resistance. As adaptation to hypoxia, changes in gene expression affecting cellular and physiological functions often result in increased cancer aggressiveness and treatment resistance [650,651,652]. A recent study using in silico modeling and the simulation of in vivo cancer cell growth found that increasing oxygen concentration and pH value in the TME could result in significant shrinkage of tumor growth size [653]. Melatonin is an effective oncostatic agent capable of modulating important elements in TME that drive immunosuppression, cell proliferation, metastasis, and resistance to apoptosis [654]. Using melatonin to maintain RBC heme redox balance and band 3 functionality directly targeting the hypoxia feedback cycle in TME could be an important linchpin in dismantling the TME to enhance drug sensitivity [655,656,657,658,659].

Hypoxic stress promotes phase separation of glycolytic enzymes into cytoplasmic G-bodies that increased glycolytic output in *S. cerevisiae* and human hepatocarcinoma cells [660,661]. Hypoxia can induce increased expression of PrP^C^ [476] to facilitate persistence and storage of memory in animals and plants [24,25,662]. In vivo and ex vivo models showed post-hypoxic cells reoxygenated in the bloodstream retained a hypoxia-induced cancer stem cell-like phenotype where exposure to intratumoral hypoxia promoted chemotherapy resistance, increased recurrence, and capacity to metastasize in post-hypoxic cells compared to cells never exposed to hypoxia [663]. The fact that pathological prion isoforms can remain dormant for an extended period of time may be another significant consideration in targeting dormancy in cancer. Cancer cells become dormant when they switch from an active to a quiescent state and cancer dormancy remains a major challenge in clinical oncology where tumor recurrence can resurface years after initial diagnosis [664]. Not surprisingly, stress has been identified as one of the triggers that can awaken cancer cells from dormancy [47,665], and hypoxic stress that reduces pH is able to activate prion aggregation [666] and phase separation (Section 2.3.3).

#### 3.5.1. Hypoxia in TME Is Modulated by Fluctuations in Red Blood Cell Flux

Band 3, or anion exchanger 1 (AE1), is probably the world’s quickest bicarbonate/chloride transporter with a turnover of ~105 chloride ions per second per molecule [667,668,669]. The C-terminal domain of this large polytopic membrane protein is embedded in the lipid bilayer, tethered to the cytoskeleton comprising the RBC membrane [670,671,672]. Band 3 is not only a critical anion transporter supporting oxygen delivery by RBCs [673], but also a primary scaffolding structure for large macromolecular complexes that modulate RBC membrane flexibility and integrity [670,674]. Disruption of band 3 and its association with proteins such as ankyrin-1 and spectrin tetramers in the RBC skeletal network [670] can induce a four-fold reduction in membrane stiffness that negatively impacts RBC membrane deformability and elasticity [675,676]. RBCs must maintain a high degree of deformability and elasticity in order to travel through capillaries and small vessels with diameters under 5 µM to fulfill their primary objective of oxygen delivery [677,678,679]. The loss of band 3 functionality can directly impact hypoxia in cancer TME, activating a positive feedback cycle where hypoxia increases band 3 disruptions to reduce RBC deformability, which in turn augments the reduction in red cell flux and O_2_ delivery.

The deformability of RBCs, which is regulated by membrane flexibility, supports the normal transit of RBCs through capillaries with lumens narrower than the cell diameter of RBCs [680,681]. Capillary RBC flux is possibly the most important determining factor for oxygen delivery to cells [682] where changes in red cell flux (RCF) can lead to changes in vascular pO_2_ resulting in transient hypoxia. Experimental studies revealed that even in well-vascularized regions of tumors, a two-fold variation in RCF can produce intermittent hypoxia (IH) in 30% of the tissues, whereas in poorly vascularized regions, the same degree of fluctuation produced significantly higher levels of transient hypoxia [683]. In addition, oxygen delivery by RBCs can be decreased by excess oxidative stress [684]. High O_2_ tension in arterial blood and hemoglobin’s natural inclination to become “biologic Fenton reagents” result in the continuous production of ROS within RBCs [621,685]. Oxidative stress, often elevated in patients with sickle cell disease (SCD), was found to be associated with increased hemoglobin degradation, which correlated negatively with decreased RBC deformability [686,687]. SCD is caused by a substitution of valine for glutamic acid at the β-6 position in the hemoglobin β-chain [688,689]. This polymorphism constrains band 3 mobility impacting RBC membrane properties [690] which not only decreases RBC deformability but also affects the ability of RBC to lower oxidative stress. Since erythrocytes lack the TCA cycle, the only source for the reducing equivalent NADPH that recycles oxidized glutathione (GSSG) to GSH is the pentose phosphate pathway (PPP) [691,692]. In erythrocytes, PPP facilitates the continuous reduction of NADP^+^ to NADPH via the conversion of glucose 6-phosphate (G6P) to 6-phosphogluconolactone catalyzed by glucose-6-phosphate-dehydrogenase (G6PD) [693]. Under steady-state conditions, the main G6P flux is maintained via glycolysis. However, the flux to PPP under oxidative stress can be enhanced more than 20 times [694], and band 3 plays a critical role in the maintenance of glycolytic flux to PPP in RBCs.

#### 3.5.2. Hypoxia Prolongs Deoxygenation and Elevates Hemin Release to Damage RBC Membrane Integrity and Band 3 Proteins

Glycolysis in RBC is responsible for the production of NADH [695], which is used by CYB5R3 to reduce MetHb [629], and the deoxygenation of erythrocytes (deoxyHb) can increase glycolysis by 26% in RBCs [696]. During deoxygenation, the temporary dissociation of ankyrin from band 3 that releases the spectrin/actin cytoskeleton from RBC membranes can improve blood flow by enhancing RBC deformability without a loss in elasticity [677,697]. However, hypoxia can increase deoxyHb [698] to prolong deoxygenation, rupturing band 3-ankyrin bridges to decrease membrane mechanical stability, deformability, increase abnormal morphology, and induce spontaneous vesiculation of RBCs [697,699]. Under normal oxygenation and deoxygenation conditions, band 3 suppresses glycolytic flux to maintain pentose phosphate pathway activities by forming complexes with glycolytic enzymes (GEs), inhibiting glycolysis. However, when oxygenated RBCs were treated with pervanadate, a reagent that inhibits band 3 protein binding by inducing phosphorylation of tyrosines [700], glycolytic fluxes were increased by 45% while PPP shunt fluxes became 66% lower than controls as a result of GE-band 3 complex inhibition [701]. Regardless of oxygenation status, GEs in band 3 knockout mice are unable to bind to RBC membranes but are distributed throughout the cytoplasm [702]. Interestingly band 3 regulates its own phosphorylation according to stress sensed in the environment.

Band 3 has been proposed to be a “redox stress sensor” that regulates its own phosphorylation as an adaptation to stress via dissociation from ankyrin and the spectrin-actin skeleton, which alters membrane structures [703,704]. Even though band 3 can selectively phosphorylate and remove oxidized regions from RBC membranes [705], increased hemin release as a result of oxidative stress [706] may still impact RBC deformability, decreasing O_2_ delivery [693]. Hemin has been shown to cause rapid destruction of RBC membrane integrity by destabilizing spectrin–protein 4.1–actin interactions [706]. Protein 4.1, a principal constituent of RBC membranes, can be mobilized in a dose-dependent manner to cause complete loss of ankyrin-band 3 binding at high hemin levels [707,708]. In addition, hemin aggregates bound to RBC membranes can reorganize membrane lipid composition to induce membrane disorder and permeabilization [709].

#### 3.5.3. Oxygen Saturation and Transport Are Directly Modulated by Heme Redox Balance

The oxidative state of MetHb (Fe^3+^) also shifts the oxygen dissociation curve to the left, where the conversion of a ferrous atom to the ferric state results in increased affinity of the remaining ferrous atoms for O_2_, thus negatively impacting O_2_ transport and release [501,710,711]. On the other hand, band 3 may act as a “molecular switch” that mediates O_2_ transport by modulating O_2_ saturation and erythrocyte properties [712,713]. The preferential binding of band 3 to deoxyHb shifts the O_2_ dissociation curve to the right in a concentration-dependent manner [714]. Kidney band 3 proteins lacking residues that bind deoxyHb were unable to alter Hb-O_2_ affinity [715]. Therefore, maintaining heme–hemin redox homeostasis is a critical consideration in controlling hypoxia in TME. Early experimental results showed the PPP shunt only accounted for a small part of the reduction of total MetHb [716] and excess hemin reduction may require NADH-dependent CYB5R3 ferrous-ferric iron recycling. Melatonin has been demonstrated to enhance NADH recycling to regenerate CYB5R3 in the reduction of MetHb [641,642], protect RBC morphology, and maintain expression of band 3 [645] all in an antioxidant-independent manner. In addition, melatonin was able to shift the O_2_ dissociation curve to the right, increasing O_2_ release, in rats exposed to hypothermia [717]. Melatonin may also protect band 3 through modulation of lipid composition. It is perhaps not a coincidence that one of the important physiological functions of PrP^C^ is heme/hemin-binding, and that both band 3 and PrP^C^ reside in lipid rafts.

#### 3.5.4. The Role of Membrane Lipids and Lipid Rafts in Prion Physiological Function and Pathological Propagation

Membrane surfaces offer distinct advantages in the formation of MLOs [718,719,720]. Lipid rafts, which are phase-separated regions in membrane lipid bilayers, enable thermodynamic interactions between membrane-anchored proteins and condensate components, facilitating phase separation [721]. The unstructured N-terminal domain of PrP^C^ is intrinsically disordered and is prone to phase separation under hypoxia or other stressful conditions such as changes in pH in TMEs. The PrP^C^ GPI anchor signal comprising residues 231–253 is usually found tethered to lipid rafts [272,333,722] (Figure 1). Upon cleavage, the soluble N-terminal cleavage fragments (N1, N2) are released while the C-terminal cleavage fragments remain bound to lipid rafts on membranes via GPI anchors [272,380,381]. The shedding of PrP^C^ by proteolysis cleaves residues 23–230 from the membrane GPI anchor around residue 231, releasing a full-length, soluble PrP^C^, which was shown to reduce the cytotoxicity of amyloid-β oligomers [382]. The N-terminal domain of PrP^C^ also contains a lipid raft-targeting region that allows interactions with membrane lipids in a GPI-independent manner [272,325,326,722]. However, tethering of the N-terminal domain to lipid rafts can compromise prion protein cellular response to oxidative stress from increased aggregation of PK-resistant N-terminal fragments [723,724]. The composition of lipids in membranes and lipid rafts can influence lipid–protein interactions, which induce either the formation of α-helix structures or β-sheet-rich amyloids [383,725]. In vitro studies reported that under physiological conditions, interactions between anionic lipids and rPrP can overcome energy barriers to increase β-sheet aggregation, converting a significant portion of α-helix in soluble, full-length rPrP to a PK-resistant conformation similar to PrP^Sc^ [726]. Nevertheless, it is possible that the structured C-terminal domain contained in full-length WT PrP may be protective against formation of β-rich amyloid-like aggregates.

### 3.6. Melatonin May Prevent PrP^C^ Pathological Conversion from Phase Separation Caused by Mutations

The C-terminal domain (residues 121–230) of human prion (huPrP) was shown to undergo large conformational changes induced by reductions in pH and increases in temperature [727]. Copper bound to the fifth, nonoctarepeat binding site in the segment containing histidine residues 96 and 111 changes the structural plasticity of the N-terminal to a more compacted conformation that may facilitate prion conversion [366,728,729] (Figure 1). Simulation of the conformational transition from PrP^C^ to PrP^Sc^ using ratchet-and-pawl molecular dynamics (rMD)-based methodology revealed that the C-terminal domain acts as a primary conversion surface for the unstructured N-terminal domain, initiating a cascade of conformational transitions that provide further templating leading to the complete conversion into the pathological PrP^Sc^ isoform [730]. Even though copper-binding can affect conformational changes in the C-terminal domains to alter aggregation behavior, mutations in C-terminal domains in both yeast and human PrP can greatly influence prion propagation also [120,731].

The pathological mutation at residue 145 (Y145Stop), located within the highly structured globular C-terminal domain (121–230), produces a highly disordered region that spontaneously phase separates under physiological conditions resulting in a truncated N-terminal that lacks C-terminal fragments [120,732]. Even though mutant Prp^145^ is normally degraded rapidly by the ubiquitin-protease system (UPS), PrP^145^ is prone to aggregation and intracellular accumulation under stress or aging-related reduced proteasomal functions [120,732]. Transgenic (Tg) mice with PrP but lack C1 fragments showed accelerated accumulation of pathogenic PrP^Sc^ after scrapie inoculation, whereas Tg(C1) mice expressing N-terminally deleted forms (PrP(Δ23–111)) in the absence of endogenous PrP remained completely healthy and did not accumulate PK-resistant PrP after scrapie inoculation [733]. Even though prion proteins are believed to be the cause for neurodegenerative diseases, under physiological conditions, PrP^C^ often act as important stress-induced signaling molecules to activate neuroprotective features to counter hypoxic brain damage (rodent in vivo, human brain tissue in vitro) [734] and ischemic injury (rat model) [735], whereas the deletion of PrP^C^ in brains of transgenic PrP^C^-knockout mice increased infarct size by 200% [736] and aggravated neuronal cerebral ischemia through reduced post-ischemic phospho-Akt expression that impaired the antiapoptotic PI3K/Akt signaling pathway [737]. Since melatonin regulates the UPS and promotes ubiquitination [308,738,739,740], the presence of adequate melatonin may ensure the proper, timely degradation of mutated PrP^145^ by ubiquitin [732] to prevent phase-separated condensate formation of self-templating amyloid-like aggregates and pathological truncation of PrP^C^. Without adequate melatonin, even in the absence of mutations, the physiological association of the N-terminal lipid raft-targeting region may cause lipid and membrane disruptions that alter membrane functions, signaling, and band 3 protein functionality, which can all exacerbate the detrimental effects of TMEs to enhance MDR.

## 4. The Effects of Melatonin on Lipid Phase Transition, Lipid Composition, and Prion Propagation in Cancer Multidrug Resistance

Lipid rafts are dynamic, transient, mobile, nanoscopic (10–200 nm) liquid-ordered (L_o_) domains that are rich in sphingolipids and cholesterol formed as a result of thermodynamic LLPS [741,742]. The location of lipid rafts on plasma membranes, intracellular membranes, and extracellular vesicles enable relevant biological functions, effectively serving as hotspots for signal transduction [743], trafficking, and sorting of proteins and lipids [744,745]. However, lipid rafts are increasingly associated with cancer MDR as quite a few cancer-related proteins involved in migration, invasion, and metastasis are found in lipid rafts, which serve as signaling hubs for these proteins [328,746,747,748]. Multidrug resistance protein 1 (MDR1), one of the ATP-binding cassette transporters responsible for drug efflux, resides in lipid rafts in prostate cancer cell lines [749], and the inhibition of flotillins—scaffolding proteins that are key components in lipid rafts—was shown to reverse MDR in colon cancer cell lines [750].

Lipid rafts have been extensively studied for the localization, trafficking, cellular signaling, cell-to-cell transmission, and conversion of PrP^C^ to PrP^Sc^ [272,326,751]. Lipid rafts are also involved in the metal/copper-mediated endocytosis of prions via clathrin-coated pits or caveolae [370,371,372]. In proliferating neuronal CAD 5 cell lines, PrP^C^ is predominantly associated with lipid rafts on cytoplasmic membranes [752] while in human dental pulp mesenchymal stem cells, the integrity of lipid rafts is essential for the preservation of recombinant prion protein (23–231) physiological activities affecting neuronal differentiation and signaling. The critical localization of PrP in lipid raft microdomains allows prions to recruit and interact with important biochemical signaling partners [753,754]. Even though lipid rafts may influence the conversion of PrP^C^ into PK-resistant isoforms [723,724], interactions between PrP N-terminal residues and membranes can also lead to membrane dysfunctions [755].

The amyloidogenic prion residues 106–126 [756,757] in the N-terminal domain are characterized by hydrophilic and hydrophobic regions that can increase lipid density and membrane viscosity upon embedding into lipid bilayers [758]. The prion peptide fragment 106–126 can form heterogenous single cation channels with different conductance and kinetic properties in lipid bilayers, modifying electrolyte homeostasis and affecting cellular functions [759,760,761] while the conversion of PrP^C^ to PrP^Sc^ is often associated with membrane abnormalities including decreased membrane fluidity [762]. The conversion process of PrP^C^ to PrP^Sc^ involves the conformational change of α-helical structures to PK-resistant β-sheets rather than chemical modifications. These conformational changes dysregulated membrane receptors causing a 5-to 13-fold reduction in bradykinin (Bk) binding affinity despite a 3-to 4-fold increase in Bk receptors on neuro N2a cells resulting in decreased Ca^2+^ and Bk second-messenger IP_3_ responses [763]. Due to the amphipathic nature of the prion fragment 106–126, it has been proposed that the toxic effects resembling many membrane-active antimicrobial peptides (AMPs) are initiated by the direct association of monomeric peptides with membrane matrix. Experimental studies employing atomic force microscopy, Raman and electron paramagnetic resonance spectroscopy, revealed that PrP 106–126 membrane interactions can impair bilayer mechanical integrity via the modulation of both line tension, which can produce porous defects, and lipid vibrational dynamics. PrP 106–126 membrane interactions can enhance intra-chain conformational disorder without altering inter-chain interactions in cylindrical-shaped phosphatidylcholine lipid molecules but increase inter-chain interactions without changing the intra-chain conformational order in cone-shaped phosphatidylethanolamine lipid molecules [764].

The physiological relationship between prions and lipid membranes may be dependent upon the presence of a sufficient level of melatonin in order to prevent or ameliorate potential pathological outcomes. Infecting transgenic mice that expressed PrP without GPI anchors with a stable form of PrP^Sc^ produced a completely new prion strain with 25–50-times higher levels of PK-resistant PrP^Sc^ compared to WT mice. However, C57BL/6 mice were selected for the breeding of GPI-knockout mutants used in these experiments [765]. Most inbred mice including C57BL/6 exhibit reduced melatonin production where the serotonin N-acetyltransferase (arylalkylamine N-acetyltransferase, AANAT) mRNA encodes a severely truncated AANAT protein due to a stop codon being spliced into a pseudo-exon, with the C57BL/6J strain exhibiting complete melatonin “knockdown” [766], whereas two very short peaks in the middle of darkness and at light onset were observed in C57BL/6 mice [767]. It is, therefore, not unreasonable to hypothesize that the lack of continuous presence of melatonin in plasma lipid bilayers contributed to the pathogenic conversion of PrP fragments interacting with lipids in membranes.

### 4.1. Melatonin Maintains Lipid Raft Integrity and Prion Physiological Functions by Modulating Cholesterol and Lipid Phase Transitions

The amyloidogenic PrP106–126 residues exhibit fusogenic properties, promoting lipid mixing [768] which can be exacerbated by low pH or high cholesterol levels [769,770,771]. In fact, cholesterol suppression has been shown to mediate prion propagation where PrP^C^ degradation and PrP^Sc^ conversion were substantially reduced in cholesterol-rich neuronal N2a cells treated with lovastatin, an inhibitor of the rate-limiting enzyme in the 3-hydroxy-3-methyl-glutaryl-CoA (HMG-CoA) reductase cholesterol biosynthetic pathway [772,773]. In addition, PrP106–126 membrane interactions can impair bilayer mechanical integrity to form pores via modulating line tension [764]. Line tension maintains the energetic boundaries between lipid raft domains and surrounding membranes, and can, therefore, affect the physiological size, form, and shapes of lipid rafts [774]. Increasing cholesterol content in membrane lipids can reduce line tension to produce nanoscopic lipid rafts [775], which, theoretically, is a desirable physiological state as opposed to enlarged, micron-sized lipid rafts that are produced under inflammatory conditions and carry pro-inflammatory, oncogenic signaling molecules [328,776,777]. However, in a highly oxidative, low pH TME, the effect of cholesterol on lipid rafts and prion propagation in the absence of adequate melatonin as a “broad-based metabolic buffer” to regulate lipid peroxidation, line tension, and cholesterol homeostasis becomes highly questionable (Figure 2).

Breast cancer and prostate cancer are associated with high serum cholesterol [778,779] while their respective cell lines have been shown to contain more lipid rafts that were sensitive to cholesterol depletion-induced apoptosis compared to healthy cells [780]. Cholesterol metabolism is increasingly associated with cancer MDR from increased gene transcription of drug efflux transporters or reprogramming of metabolic pathways that enable MDR phenotypes [781,782,783]. Depletion of cholesterol in lipid rafts in drug-resistant cancer cells was demonstrated to facilitate the accumulation of doxorubicin or rhodamine 123 via suppressing MDR-1 activity and increasing drug sensitivity to overcome drug resistance [784]. Increased cholesterol, on the contrary, can facilitate prion propagation due to its inherent electrostatic properties. Under physiological conditions, interactions between anionic lipids and rPrP can overcome energy barriers to increase β-sheet aggregation, converting a significant portion of α-helix in soluble, full-length rPrP to a PK-resistant conformation similar to PrP^Sc^ [726]. Increasing cholesterol content can lower surface charge of lipid membranes in saline solutions from positive to negative [785]. Therefore, excess cholesterol may alter prion interactions with negatively charged anionic lipids to intensify the aggregation of PK-resistance β-sheet amyloids [786,787].

Melatonin not only directly interacts with cholesterol to counteract and alleviate the effects of cholesterol on lipid membranes [788], but also regulates lipid dynamics and composition, inducing lipid phase separation by modifying lipid hydrocarbon chain order [330,331]. By increasing disorder in the L_d_ phase, melatonin displaces cholesterol, driving cholesterol into the ordered L_o_ phase via competitive binding to lipid molecules [330]. The preferential location of melatonin at hydrophilic/hydrophobic membrane interface due to its ability to form strong H-bonds with hydrophilic lipid headgroups allows nonpolar melatonin to reverse cholesterol- and prion-induced membrane rigidity [762,789,790,791,792,793]. In the POPC/bovine brain sphingomyelin-supported lipid bilayer and POPC/bovine brain sphingomyelin/cholesterol-supported lipid bilayer membrane models, the PrP106–126 fragment was demonstrated to cause membrane thinning in the L_o_ phase and membrane disintegration in the L_d_ phase [329]. More importantly, the results obtained suggest that PrP106–126 fragment membrane interactions mainly occurred in the L_d_ phase where the peptides bound to the headgroup region of lipids in the L_d_ phase of the membrane increased membrane strain [329]. Since melatonin can stabilize lipid L_o_/L_d_ phase-coexistence over an extended range of temperatures (up to 45 °C), effectively preventing the formation of the L_d_ phase at high temperatures [332], it is quite possible that local variations in melatonin concentration can affect prion interactions with membrane lipids via the reordering of membrane lipids, which impacts the lipid phase transition, line tension, membrane fluidity, and functionality of lipid rafts.

At 0.5 mol% concentration, melatonin can penetrate lipid bilayers to form fluid domains where melatonin molecules are aligned parallel to phospholipid tails, but at 30 mol% concentration, melatonin molecules become aligned parallel to the lipid bilayer close to the headgroup regions where one melatonin molecule associates with up to 2 lipid molecules, forming an ordered, uniform, lateral, crystal-like structure evenly distributed throughout membrane models tested [794]. The fact that exogenous melatonin supplementation injected at doses between 10 and 200 mg/kg showed dramatically different dose-dependent subcellular distribution in male Wistar rat cerebral cortex, where membranes were able to reach 10-times higher concentration levels than in the cytosol [795], may imply that high melatonin concentration produced under duress may act as a “broad-based metabolic buffer”, disrupting prion interactions with membrane lipids to prevent aberrant phase separation resulting in pathological aggregations [721,726] while defending band 3 proteins from membrane disruptions caused by prion-hemin binding effects (Figure 2).

### 4.2. Melatonin May Preserve Band 3 Interactions with Membrane Lipids in Antioxidant-Dependent and -Independent Manners

Results from more recent atomistic molecular dynamics (MD) simulations investigating interactions between band 3 and nanoscopic lipid raft domains support early experimental observations that band 3 prefers to localize in L_o_ lipid raft domains albeit the concentration of cholesterol, comprising ~45 mol% of erythrocyte membranes, greatly affects membrane and band 3 interactions [796,797,798,799,800]. Cholesterol enrichment resulting in an elevated cholesterol-to-phospholipids mole ratio exceeding the normal 0.9–1.0 amount resulted in decreased membrane fluidity and strikingly abnormal changes in red cell contours characterized by deranged folding and scalloping of cell margins [800]. These changes may be explained by band 3 interactions with lipid phases as a result of cholesterol enrichment. All-atom MD simulations revealed that in ternary lipid bilayers composed of saturated lipids, unsaturated lipids, and cholesterol, the band 3 C-terminal domain, which is associated with the erythrocyte cytoskeleton, interacted with high electrostatic attraction with anionic lipids in the L_o_ domains of phase-separated lipid bilayers, whereas in lipid bilayers with increased cholesterol concentration (50 mol%), band 3 was observed to preferentially target the L_d_ phase and avoided contacts with cholesterol-enriched L_o_ domains [796]. The association of band 3 with lipid nanodomains in erythrocytes greatly influences physiological functions [667,801] where band 3 fragments can move into lipid bilayers, seeking each other out to form functional fragments [802,803]. Thus, disturbances in lipid composition as a result of hypoxia or increased ROS in TMEs can potentially disrupt band 3 and associated proteins, including ankyrin-1 and spectrin tetramers in erythrocyte skeletal networks [670,697,699], reducing RBC membrane deformability and elasticity [675,676] and lowering O_2_ saturation [498] to reinforce the negative feedback, which enhances TME-induced MDR.

Lipid peroxidation is a cascading event initiated by ROS attacking anionic headgroups at membrane interfaces [804] where oxidized moieties residing close to lipid headgroups perturb membrane bilayer structures, modifying membrane properties including increasing membrane permeability [805], decreasing membrane fluidity [806,807], and increasing line tension, which can transform nanometer-scale lipid rafts into larger, micron-sized domains [776,808,809] that carry pro-inflammatory molecules often associated with cancer cell signaling pathways [328,777,780]. Experimental results using giant membrane vesicle model systems showed that lipid peroxidation induced significant changes in membrane phase behavior, causing a dramatic escalation of phase separation at room temperature, which increased the non-raft phase while decreasing affinity of tested raft proteins for raft domains [810]. Melatonin, with its free radical scavenging metabolites [131,133,134] and preferential location in membrane bilayer headgroups, enables dynamic interactions that can attenuate peroxidation effects via a reduction in bilayer thickness and increasing fluidity [790,794,811] while the presence of both hydrophilic and lipophilic moieties facilitates the neutralization of both aqueous and lipophilic free radicals including hydroxyl radical (^•^OH) and hydroperoxyl radical (^•^OOH) [126,812,813]. The fact that melatonin prevents lipid peroxidation cascades and stabilizes lipid L_o_-L_d_ phase separation over a range of temperatures to prevent the formation of non-raft L_d_ phase become especially meaningful when reports from correlative studies identified lipid peroxidation as the primary pathogenic event associated with the propagation of PK-resistant PrP^res^ converted from physiological PrP^C^ [814]. Hence, without viable prions to contain damages of free hemin caused by excess oxidative stress, hemin aggregates bound to RBC membranes can reorganize membrane lipid composition to induce membrane disorder and permeabilization [709].

It is apparent that the role of melatonin in attenuating lipid peroxidation, preserving lipid raft and band 3 integrity, and supporting prion physiological functions can effectively terminate negative feedback influences that exacerbate MDR in TMEs. Even though melatonin has been demonstrated to both increase and inhibit prion expression, the seemingly controversial pleiotropic features of melatonin only accentuate its ultimate functions acting as a “broad-based metabolic buffer” that can support prion physiological stress-response functions but suppress pathological, self-templating aggregates activated by hypoxic, stress-laden TMEs (Figure 2).

### 4.3. The Pleiotropic Effects of Melatonin in the Regulation of Prions in Cancer Multidrug Resistance

Various experimental studies demonstrated that low levels of melatonin upregulate the expression of PrP^C^, stimulating antioxidant, protective, survival responses. Both in vitro and in vivo studies reported that melatonin upregulated the expression of PrP^C^ to rescue mesenchymal stem cells (MSCs) from oxidative stress-induced apoptosis at only 1 μM concentration [113], whereas silencing of PrP^C^ inhibited all melatonin-mediated therapeutic effects on MSC proliferation and functionality at the same 1 μM dose [118]. A quantity of 1 μM melatonin co-administered with 5 μM pioglitazone not only prevented indoxyl sulfate-induced senescence but also promoted high growth rates in MSCs [647]. Treatment of human renal proximal tubule epithelial (TH1) cells with 1 μM melatonin increased expression of PrP^C^ to augment antioxidant effects against high glucose-mediated fibrosis, successfully preventing fibrotic phenotype changes [648] (Table 1). Alternatively, in order to inhibit or reverse prion-mediated oncogenic effects and drug resistance, a much higher dosage is often used instead.

In a high oxidative TME, adequate NADH may be necessary to serve as essential substrates for CYB5R3 to reduce MetHb from ferric to ferrous heme [629,630]. Experimental studies showed that at 2 mM concentration, melatonin provided the greatest protection against loss of NADH from exposure to 500 µM orthovanadate (Va^5+^) incubation [642], whereas to reverse acid pH_e_, a lower but continuous presence of melatonin was demonstrated to be requisite [489]. Hence, even though 1 mM melatonin was able to significantly reduce in vitro proliferation and migration in murine melanoma B16-F10 cells, in vivo B16-F10 murine models using C57BL/6J mice treated with melatonin at 20 mg/kg (intraperitoneal injection or drinking water) matching in vitro dosage were unable to prevent metastasis or curb proliferation [649] (Table 1). C57BL/6J are inbred mice expressing severely truncated AANAT that results in complete melatonin “knockdown” [766]. It is possible that 20 mg/kg melatonin supplementation via intraperitoneal injection or drinking water in murine models incapable of producing melatonin did not provide a continuous presence of melatonin required to reverse acidic pH_e_ conditions that promote melanoma metastasis and proliferation [649,815]. However, 1 mM melatonin did promote colorectal cancer cell apoptosis by decreasing expression of PrP^C^ and PINK1 to increase superoxide accumulation resulting in mitochondria-mediated cell death. The effects of melatonin were amplified when PrP^C^ was completely knocked down [114]. By contrast, in a non-cancerous environment, melatonin upregulated PrP^C^ and PINK1 where MSC harvested from chronic kidney disease mouse models treated with 100 μM melatonin exhibited reduced H_2_O-induced senescence compared to normal mouse MSC [113] (Table 1).

The interactions between prions and lipid rafts may also affect cancer stem cell regulation. Lipid rafts and caveolae play important roles in maintaining the self-renewal of embryonic stem (ES) cells by facilitating receptor-mediated signal transductions [816,817]. Cancer cells and ES cells share common gene transcription regulators such as Oct4 which contributes to pluripotency [818,819,820]. PrP^C^ is able to regulate cancer stem cell properties via interactions with stem cell marker proteins [66]. By interacting with human mesenchymal-epithelial transition factor (c-MET), PrP^C^ upregulated Oct4 to enhance cancer stem cell characteristics in colorectal cancer [821]. In various specimens from colorectal cancer patients, PrP^C^ was found to directly regulate Oct4, and the expression of PrP^C^ and Oct4 were both upregulated and correlated significantly with metastasis and tumor stages [115]. Melatonin, not surprisingly, can enhance drug sensitivity, inhibiting colon cancer progression by regulating PrP^C^ interactions with Oct4. Treating human colon cancer stem cells (CSCs) with 500 μM melatonin and 1 μM 5-fluorouracil (5-FU) caused apoptosis and inhibited expression of the stem cell marker Oct4 by inhibition of PrP^C^ expression [115]. When used in combination with 1 μM oxaliplatin, 500 μM MEL melatonin promoted apoptosis of oxaliplatin-resistant colorectal cancer cells, again, by inhibition of PrP^C^ [116]. Overexpression of PrP^C^ plays a vital role in colorectal cancer oxaliplatin-resistance via enhanced superoxide dismutase (SOD) and catalase antioxidant activities, and oxaliplatin-resistance cancer cells often exhibit reduced intracellular superoxide anion generation. In addition, by inhibiting PrP^C^ expression, melatonin can induce endoplasmic reticulum (ER) stress and apoptosis in oxaliplatin-resistant cells, effectively blocking oxaliplatin-associated elevation of SOD and catalase antioxidant activities [116] (Table 1).

The use of melatonin at appropriate levels relevant to context may be a principal consideration when targeting prion and associated biological functions such as stem cell regulation. In non-cancerous settings, melatonin has been demonstrated to enhance pluripotency and stem cell proliferation at 500 ng/L (2.15 nmol/L) by inducing the expression of stem cell markers including Oct4 [822]. At 10 nmol/L concentration in human non-cancer cells, melatonin selectively upregulated transcription of pluripotency and differentiation markers such as NANOG [823], completely contrary to findings in ovarian cancer stem cell experiments where melatonin inhibited the invasion and migration of cancer stem cells by inhibiting NANOG expression, albeit at exceptionally high concentrations between 3.4 and 6.3 mM [824]. It is tempting to hypothesize that at higher concentrations, melatonin can modulate inhospitable environment to attenuate PrP^C^ stress responses, whereas lower levels stimulate and support the natural, physiological protective activities of prions.

## 5. Conclusions

Liquid–liquid phase separation is postulated as the fundamental process driving the formation and dissolution of biomolecular condensates as rapid, energy-efficient, adaptive survival responses to exogenous and endogenous stress. Melatonin and prions are both ancient, evolutionarily conserved molecules exhibiting synergistic relationships that are integral to the stress response pathways employed ubiquitously by living organisms to counter exogenous and endogenous stress. Aberrant phase separation resulting in the aggregation of condensates may be implicated in the conversion of prions from physiological soluble isoforms to pathological, self-templating isoforms intended to enhance survival via non-Mendelian, epigenetic inheritance, which, ironically, may enhance cancer drug resistance in less-than-optimal tumor microenvironments. As a “broad-based metabolic buffer” in a highly-stressed TME, melatonin can not only temper pH and oxygen imbalances to support PrP^C^ physiological functions and prevent phase separation-induced pathological aggregation and conversion, but may also modulate epigenetic adaptations promoting metastasis, invasion, and stemness by intervening heme-and membrane-PrP^C^ interactions via redox activities and lipid homeostasis and lipid phase transition stabilization, respectively. If the in silico observation of increased expression of PrP^C^ in cancer cells under optimal conditions does not modulate proliferation, resistance to cell death, and metabolism can be independently confirmed by in vitro/in vivo studies, then the concept of melatonin as a “broad-based metabolic buffer” characterized by exceptional antioxidant-dependent and -independent features that can fine-tune the tumor microenvironment at appropriate or even continuous applications may be an additional, but perhaps essential, consideration as a viable therapeutic solution to counter cancer MDR.

## Figures and Tables

**Table 1:** Pleiotropic effects of low and high melatonin doses on in vitro and in vivo models involving prion propagation and/or associated processes.

Model/Description	Melatonin Doses	Melatonin’s Effects	Reference
MSCs/Model of ER stress–induced ischaemic injury.	1 μM MEL pretreatment 30 min at 37 °C.	Increased expression of PrP^C^ and antioxidant enzymes to reduce oxidative stress.	[113]
MSCs/Model of indoxyl sulfate-induced senescence.	1 µM MEL + 5 µM pioglitazone.	Treatment promoted highest MSC growth rates and inhibited senescence via enhanced PrP^C^ expression.	[647]
TH1/Model of high glucose-mediated fibrosis.	1 µM MEL as pretreatment.	Prevented high glucose-induced fibrosis by recovering PrP^C^ expression to augment antioxidant protection.	[648]
SNU-C5/WT cells/Model of colorectal cancer cell apoptosis.	1 mM MEL treatment 24 h.	Reduced PrP^C^ and PINK1 expression to increase mitochondrial superoxide.	[114]
Human colon CSCs (S707)/Model of PRNP overexpression.	500 μM MEL + 1 μM 5-FU treatment for 72 h.	Treatment suppressed proliferation and increased apoptosis by inhibiting PrP^C^-OCT4 axis.	[115]
Murine/Model of human CSCs (S707) xenograft tumorigenesis.	500 μM MEL + 1 μM 5-FU treatment for 72 h.	Treatment decreased PrP^C^ expression to reduce tumor volume and suppress cell proliferation.	[115]
SNU-C5/Oxal-R/Model of PrP^C^ expression in oxaliplatin-resistant colon cancer cells.	500 μM MEL + 1 μM oxaliplatin for 24 h.	MEL induced oxaliplatin-mediated apoptosis via blockade of PrP^C^-mediated antioxidant activities.	[116]
PC12/Model of paraquat-induced NADH depletion.	1 mM MEL incubation at 35 °C for 1 h.	Prevented the loss of NADH/NAD+ caused by paraquat treatment.	[642]
Oxyhemoglobin/Model of vanadate-induced NADH oxidation.	2 mM MEL.	Treatment conferred the highest level of protection against NADH oxidation compared to lower doses.	[642]
Murine/Model of B16-F10 melanoma cell proliferation.	1 mM MEL 24 h I incubation.	Significantly reduced growth rate and migration.	[649]
C57BL/6J mice/Model of lung metastasis via B16-F10 cell injection.	20 mg/kg in drinking water or IP injection for 15 days.	Melatonin did not alter cell migration or proliferation.	[649]
Kunming mice/Model of copper-induced liver injury.	50 mg/kg IP injection once daily, 3 times.	Inhibited copper-induced hepatotoxicity and DNA damage via copper chelation, preventing formation of hydroxyl radical.	[430]

MSC: mesenchymal stem cell; ER: endoplasmic reticulum; TH1: human renal proximal tubule epithelial cell line; SNU-C5/WT: wild-type colon cancer cell line; PINK1: PTEN-induced kinase 1; CSCs: cancer stem cells; 5-FU: 5-fluorouracil; OCT-4: octamer-binding transcription factor 4; SNU-C5/Oxal-R: oxaliplatin-resistant colon cancer cell line; PC12: adrenal phaeochromocytoma cell line; C57BL/6J mice: inbred strain with complete melatonin “knockdown”; IP: intraperitoneal; Kunming mice: outbred stock with no known report of melatonin “knockdown” (see Abbreviations for additional acronyms).

## Data Availability

Not applicable.

## Abbreviations

3OHM	3-hydroxymelatonin
Aβ	β-amyloid peptide
Aβo	amyloid-β oligomers
Akt	protein kinase B
ATP	adenosine triphosphate
COX	cytochrome c oxidase
CYB5R3	NADH-cytochrome b5 reductase 3
DNA	deoxyribonucleic acid
ER	endoplasmic reticulum
ES	embryonic stem
G6P	glucose 6-phosphate
G6PD	glucose-6-phosphate-dehydrogenase
Ga	giga annum (billion years)
GLUT1	glucose transporter 1
GOE	great oxidation event
H^+^	hydrogen proton
H_2_O_2_	hydrogen peroxide
IDR	intrinsically disordered region
L_d_	liquid disordered
L_o_	liquid ordered
LLPS	liquid–liquid phase separation
mM	millimolar
μM	micromolar
MD	molecular dynamics
MetHb	methemoglobin
MLO	membraneless organelle
MSC	mesenchymal stem cell
NAD^+^	nicotinamide adenine dinucleotide
NADH	nicotinamide adenine dinucleotide hydrogen
NLRP3	NLR pyrin domain containing 3 (inflammasome)
nM	nanomolar
^•^OH	hydroxyl radical
^•^OOH	hydroperoxyl radical
OXPHOS	oxidative phosphorylation
pH_e_	extracellular pH
pH_i_	intracellular pH
PI3K	phosphoinositide 3-kinase
POPE	1-palmitoyl-2-oleoyl-sn-glycero-3-phosphatidylethanolamine
POPC	1-palmitoyl-2-oleoyl-sn-glycero-3-phosphatidylcholine
PTM	post-translational modification
RBC	red blood cell
RCF	red cell flux
Redox	oxidation-reduction
RNA	ribonucleic acid
RNP	ribonucleoprotein
ROS	reactive oxygen species
UPS	ubiquitin-protease system
UVR	ultraviolet radiation
VDA	vascular disrupting agent
WT	wild-type
